# Changes in respiratory and cardiovascular morbidity air pollution risk and attributable burden in New York City

**DOI:** 10.1097/EE9.0000000000000497

**Published:** 2026-07-21

**Authors:** Ariel Spira-Cohen, Rebecca Goldberg, Sarah Johnson, Masha Pitiranggon, Kazuhiko Ito

**Affiliations:** New York City Department of Health and Mental Hygiene, Bureau of Environmental Surveillance and Policy, New York, New York

**Keywords:** PM_2.5_, NO_2_, O_3_, air pollution, asthma, cardiovascular disease, health impact assessment, emissions reductions

## Abstract

**Background:**

Air quality in New York City has improved substantially in the past 2 decades due to reductions in both transported aerosols and local emissions. We estimated the short-term risk changes for three criteria air pollutants and corresponding attributable burdens.

**Methods:**

Trends in percent excess risk (PER) per same pollution increment and attributable counts and fractions for fine particles (PM_2.5_), nitrogen dioxide (NO_2_), and ozone (warm season only) were examined for Emergency Department (ED) visits (2005–2019) and hospitalizations (2000–2019) for youth asthma (ages 5–17), adult cardiovascular disease (CVD) (ages 40+), and older adult respiratory causes (ages 65+). Time-series models considered up to 3-day lag, adjusted for seasonal trends, day of week, holidays, and concurrent and delayed temperature.

**Results:**

Most pollutant-outcome pairs did not exhibit trends in PERs over time. NO_2_-attributable burden was higher than PM_2.5_ burden for asthma and CVD outcomes. We estimated about 4500 (17%) youth asthma ED visits and 1000 (2%) CVD hospitalizations on average annually (2005–2019) attributable to NO_2_ compared with 1900 (7%) youth asthma ED visits and 450 (1%) CVD hospitalizations attributable to PM_2.5_. NO_2_- and PM_2.5_-attributable health impacts declined about 50%–70%, driven by decreasing pollution levels and total morbidity burden depending on outcome, pollutant, and season. Ozone-attributable burden was mostly unchanged over time.

**Conclusion:**

With less decline and greater attributable impact than PM_2.5_, NO_2_ is increasingly important for measuring morbidity impacts from local sources (i.e., traffic and buildings) in New York City. Ozone impacts from regional source emissions persist.

**What this study adds**

Past studies that investigated the accountability of emission reductions relied on natural experiments with a sudden emission reduction leading to an improvement in air quality and health impacts, but less is known about the health impacts of emission reduction that took place over longer durations (e.g., decades). In this study, we analyzed the short-term relationships between air pollution and asthma, respiratory, and cardiovascular morbidity outcomes in New York City, where multiple emission regulations and market-driven fuel type changes led to substantial reductions in ambient PM_2.5_ and NO_2_ levels over 2 decades. By characterizing limited changes in short-term risks over time and estimating attributable burdens, we show substantial health benefits associated with the air quality improvement and identify the need to further address local combustion sources of NO_2_.

## Introduction

Global efforts to inform policy decisions impacting air quality have increasingly relied on air pollution health risk assessment methods, which include health impact assessment (HIA) tools to project expected health benefits from proposed regulatory or programmatic scenarios.^1,2^ The approach has been used to assess the health co-benefits of air pollution reductions from climate mitigation strategies.^3–5^ HIAs use published concentration–response (CR) functions to assess attributable burden, which can include the estimation of costs associated with loss of life and costs of medical care.^3,6^ Some level of uncertainty is inherent in the estimates produced based on the generalizability of the CR functions to new scenarios. Factors that can play a role in uncertainties include (1) the stability of the risk estimates over time, (2) their suitability to the locale and population of interest, and (3) differences in health outcome definitions.

While HIA is generally a prospective decision-making tool, equally important is a retrospective approach where the health impact of a previously implemented policy or event (e.g., coronavirus disease [COVID] pandemic, Olympic games) results in a sudden change in emissions—known as accountability studies.^7^ As many emission regulations are phased in over time and take several years to complete, the accountability of emission reduction leading to reduced adverse health impacts cannot always be tested in these “natural experiments.”^8,9^ Rather than conceptualizing an accountability “chain,” some have suggested an accountability “web,” which highlights the myriad external forces leading to a more complex relationship of a policy’s impact on health outcomes, including changes in characteristics of the population, such as those driven by social factors and healthcare access, as well as changes in environmental co-exposures.^10^ This complexity underscores the importance of investigating the stability of CR functions over time and the impact of changes in exposure levels and outcome counts on the attributable burden.

Multiple recent studies have investigated the changes in the short-term relationships between air pollution and mortality over time, including a recent study, the largest of its kind to date, that analyzed 380 urban areas from 24 countries spanning 22 years (1995–2016).^11^ They found that, while air pollution levels over 2 decades generally declined in these cities, the estimated percent excess risks (PERs) for all-cause deaths did not change significantly over time. For morbidity outcomes, Chen et al. analyzed the trend in the short-term associations between PM_2.5_ and respiratory and circulatory hospitalizations among US Medicare beneficiaries (ages at or above 65 years) in 173 counties from 1999 to 2016.^12^ They found no significant temporal changes in the short-term associations between PM_2.5_ and circulatory hospitalizations but reported that the association for respiratory hospitalizations decreased from 1999 to 2008 and then increased by 2016. A follow-up of the Medicare data analysis expanded the study areas to 968 counties for the years 2000 through 2016 and found a similar pattern of decrease-then-increase in PERs for PM_2.5_ and respiratory hospitalizations, but they reported an increasing trend in PER for PM_2.5_ and circulatory hospitalizations.^13^ The authors speculated that potential impacts of the 2008 recession and changes in PM_2.5_ chemical components during the study period could be the cause of these changes in PERs of PM_2.5_ over time.

For New York City (NYC), our analysis of nonexternal mortality and nitrogen dioxide (NO_2_) and ozone (1990–2019) and PM_2.5_ (2000–2019) found no discernable trend in PERs for these pollutants.^14^ Researchers from New York State published a series of studies for New York State (including NYC) focusing on the associations between PM_2.5_, its source-apportioned constituents, and cardiovascular and respiratory morbidity outcomes.^15–18^ They examined the impacts of emission regulations by dividing the study period into subsets of years (e.g., 2005–2007; 2008–2013; and 2014–2016) to test if PERs of PM_2.5_ or its constituents changed after regulations were implemented. While they reported PER reduction for some morbidity outcomes (e.g., cardiovascular hospitalizations),^15^ they also observed an increase in PER for other outcomes for the more recent period.^18^ Because the impact of specific emission regulation may lag after the phase-in period,^19^ examining relatively short, hypothesized time windows may be challenging.

The objectives of this study are three-fold: (1) characterize changes in air pollution morbidity risk from short-term exposure to three criteria air pollutants of health importance (PM_2.5_, NO_2_, and ozone) in NYC; (2) quantify the relative pollution-attributable respiratory and cardiovascular morbidity burden as measured by hospitalizations and Emergency Department (ED) visits in NYC by pollutant-outcome pair; and (3) describe the trends in pollution-attributable burden over time. The last two decades saw large declines in PM_2.5_ concentration and changes in composition, most notably in the greatly diminished regional sulfate component, which prior to 2012 was the largest single PM_2.5_ constituent.^20^ Prior HIAs of PM_2.5_ and ozone in NYC^21–23^ used cardiovascular^24^ and asthma^25^ hospitalization CR functions generated before numerous regulatory and market-driven changes in building fuel types, vehicle emissions controls, and regionally transported aerosols.^20^ An update to these local risk estimates will help validate current HIA methods used for policy and planning purposes in one of the most populated cities in the United States and inform HIAs for other urban areas experiencing similar declines in pollution, particularly in ambient PM_2.5_.

## Methods

### Overview of study design

The first aim of this study is an extension of our work to study trends in short-term mortality risk in NYC, published in Goldberg et al., using a comparable methodology to examine morbidity risk trends over time.^14^ Similarly, we used time-series models to evaluate short-term associations of daily citywide average air pollution concentrations for three criteria air pollutants (PM_2.5_, NO_2_, and ozone) with ED visits and hospitalizations for youth asthma (ages 5–17), older adult respiratory causes (ages 65+), and adult cardiovascular disease(CVD) causes (ages 40+). To study the change in magnitude of risk associated with these pollutants over time, the models were run in rolling 5-year segments through 2019—starting from 2000 for inpatient hospitalizations and 2005 for ED visits, when these data became available. This approach is advantageous over fixed time windows in that it both allows for the assessment of any individual year’s influence on the PERs and is also equivalent to fixed nonoverlapping time windows at every fifth point. Seasonal models were evaluated based on seasonal differences in dominant emission sources in NYC (e.g., residual oil heating presence in winter but not summer).^20^ We also report the pollution-attributable fractions and counts from time-series models for which positive associations were found for the 15-year period when both ED visits and hospitalization data were available (2005–2019) and for which there was no observed consistent increase or decrease in risk over the 15 years or risk was close to zero. We compared the attributable burden over time and among the pollution-outcome pairs studied. Results reported in the main text focus on full-year impacts from NO_2_ and PM_2.5_ and warm-season impacts of ozone, with seasonal results included in Supplemental Material https://links.lww.com/EE/A437. All analyses were run in R version 4.2.2 (Vienna, Austria).^26^ ChatGPT-5 OpenAI (release date: August 7, 2025) was used to iteratively edit R syntax for manuscript figures. This research was approved by the New York City Health Department’s Institutional Review Board protocol number 14-036. This study involved analysis of administrative health data and met the requirements for a waiver of the informed consent process.

### Air pollution and weather data

Citywide daily average concentrations of NO_2_, ozone, and PM_2.5_ were computed from all monitors within the five boroughs of NYC and monitors in New Jersey counties that are adjacent to NYC using data from the United States Environmental Protection Agency’s (EPA) Air Quality System,^27^ adjusting for mean and variance.^28^ The monitors used were required to have a population exposure monitoring objective and meet EPA criteria for annual completeness. Daily measurements were excluded from monitors where hourly measurements were less than 75% complete. To produce a complete time-series for each pollutant, we included monitors in the counties in New Jersey adjacent to NYC within the same airshed. A map of all the monitors used for the citywide average computation and additional details on missing values are provided in the Supplemental Material https://links.lww.com/EE/A437. Daily mean temperature values were calculated from LaGuardia airport weather station data retrieved from the National Oceanic and Atmospheric Administration global hourly database.^29^

### Morbidity data

Records with valid NYC patient-reported zip codes from the Statewide Planning and Research Cooperative System (SPARCS) were aggregated by day for three key outcomes. The outcome definitions resulted in stable annual trends across the nationwide transition from International Classification of Diseases (ICD) version 9 Clinical Modification (ICD-9-CM) to version 10 (ICD-10-CM) in October 2015: asthma limited to ages 5–17 years (youth asthma), all respiratory causes (including asthma and other respiratory causes) among adults ages 65+ (older adult respiratory), and cardiovascular causes ages 40+ (adult CVD). We limited the age for asthma morbidity to 5–17 years because air pollution-induced asthma exacerbations have been shown to be stronger in younger people^30–32^ and this age group comprises nearly a quarter of all asthma morbidity in NYC.^33^ We could not analyze chronic obstructive pulmonary disorder (COPD) and asthma separately for adults due to the overlap of COPD and asthma after the ICD-9-CM to ICD-10-CM transition in 2015, but the overall group of ICD codes for respiratory diseases was stable across the coding transition. We limited to ages 65 and older for the respiratory cause outcomes because at this age, most of the visits were COPD and pneumonia, while the most influential seasonal contributor was influenza epidemics (which we were able to control for in our model’s seasonal terms). In exploratory analyses, we found that the 20–64 years age group had multiple seasonal peaks from both allergic factors (i.e., large spring asthma spikes) and seasonal respiratory infections, which resulted in challenges with model fit, and thus, results for these ages were excluded from this study. CVD outcomes were limited to ages 40 years and older to be consistent with previous analyses of this outcome in NYC,^24,34^ which are used to calculate attributable health burden reported on the NYC Health Department’s Environment and Health Data Portal.^35^ We also limited the records with the admission type of “emergency” or “urgent” to ensure visits could be related in time to short-term air pollution exposure. Cardiovascular-related records were defined as those with ICD-9-CM codes 393-398, 410-411, 413-414, 429-438, and ICD-10-CM codes I05-I09, I20-I23, I24-I25, I51, I60-I69 as principal diagnosis. Respiratory-related records were defined using the first two digits for ICD-9-CM codes of 46-51 and ICD-10-CM codes of J0-J9 as primary diagnosis, except for those for COPD, which followed inclusion criteria consistent with the Environmental Public Health Tracking network definition (ICD-9 490–492, 496 as the primary diagnosis or 493.2 as a primary diagnosis when 490–492, 496 is present in any other diagnosis codes; ICD-10 codes J40–J44).^36^

### Model specifications

We initially examined lags 0 through 3 days for each of the three pollutants (0 through 6 for asthma) and determined the lag structure most consistently associated with each outcome to be same-day exposure for adult cardiovascular causes, 0–2-day lags for older adult respiratory causes, and 0–3-day lags for youth asthma. We fit generalized linear regression models (GLM) with a quasi-Poisson distribution for the cardiovascular outcomes and generalized additive models with a negative binomial distribution for youth asthma and older adult respiratory outcomes due to differing levels of overdispersion of the data, with the two respiratory outcomes more substantially overdispersed. All pollutant-outcome pair models were run for the full year and by season (warm: May through September; cold: November through March; seasonal transition months of April and October were excluded) except for ozone models, which were evaluated for the warm season only. All models had two temperature adjustments—same-day and average of 3 days prior temperature with natural spline using 3 degrees of freedom each. Post hoc analyses included in Supplemental Material confirmed this was the most conservative model specification for temperature adjustment (see Figures S6––S11 https://links.lww.com/EE/A437). Other covariates included year, holidays, day of week, and natural spline of the day of study period using 7 degrees of freedom per year for the full-year models and 3 degrees of freedom for the seasonal models. These specifications allowed for adjustment for cyclical, seasonal, and within-season trends, including influenza epidemics, that vary from year to year in the daily outcome counts. Additional outcome-specific adjustments included a term for the transition from ICD-9-CM to ICD-10-CM for the older adult respiratory outcomes due to the overlapping definition of asthma and COPD in this age group, and for warm-season asthma models, two additional terms were added: (1) a 4-degree polynomial term considering up to 30 lag days after each school start date, which typically shows a peak in asthma visits, and (2) a 4-degree polynomial term considering up to 7 lag days of the percent rank of allergy syndrome as defined by allergic rhinitis (ICD-9-CM 477.9 and ICD-10-CM J30.9) in NYC Health Department syndromic surveillance data on near-real-time ED visits to account for the spring pollen peak impacts on asthma.^37^ Models were fit using the *dlnm* package with linear functional form and unconstrained lag form.^38^ We chose a linear functional form based on conclusions from U.S. EPA’s scientific review documents summarizing previous studies of short-term exposure–response relationships with morbidity outcomes (including similar respiratory and cardiovascular outcomes). There is limited evidence from these studies that the relationship departs significantly from linear,^39–41^ suggesting a linear relationship is reasonable. Additionally, our residual plots did not show evidence of nonlinearity. Rolling 5-year segmented models followed the same specifications as models covering the full time periods. To aid in the interpretation of our results, we also computed Pearson correlation coefficients for each pair of air pollutants for each of the 5-year moving time windows and correlations between each pollutant and concurrent daily temperature and the average of lag 1- through 3-day temperatures (i.e., the model covariates).

### Percent excess risk and attributable count calculations

PER was calculated from risk estimates generated by this study for each pollutant using the increment of pollution concentration frequently used in EPA’s Integrated Science Assessments (NO_2_: daily maximum of 24 1-hour measurements, 30 ppb;^41^ PM_2.5_: 24-hour daily mean, 10 μg/m^3^;^39^ ozone: daily maximum of 8-hour rolling average derived from 24 one-hour start hour measurements, 30 ppb).^40^ For youth asthma and older adult respiratory outcomes, the PERs reported are cumulative across the multiple lag days noted earlier. The *attrdl* function was applied to the models spanning 2005–2019 to compute the attributable counts and fractions for each pollutant-outcome pair, assuming a linear, no-threshold relationship.^38,42^ This approach ensured stable estimates over a 15-year period, which was supported by the generally positive PERs over time for the select pollutant–outcome pairs. Pollution–outcome pairs where there was no observed consistent increase or decrease in risk over the 15 years, or the effect size was close to zero for 2005–2019 were excluded from the analysis of pollution–attributable burden. Annual average attributable counts were calculated from the cumulative total, and trends were examined by limiting the data by time (i.e., year) and using the model for the full period (2005–2019).

### Sensitivity analysis

Sensitivity analyses included (1) single lags up to 6 days for youth asthma outcomes and including multiple lags up to 3 days; (2) using 8 and 4 degrees of freedom per year for natural spline of study days to adjust for seasonal trend in full-year and seasonal models, respectively; (3) different months to define warm season for asthma models due to seasonal peaks in asthma healthcare utilization in May and September, so these months were excluded singularly as well as together (i.e., May–Aug, June–Aug, June–Sept); (4) a co-pollutant as an additional term in the model; and (5) using the PER estimates from the 5-year moving window analysis to calculate attributable counts. In addition, we used the risk estimates from our time-series models and input these into the U.S. EPA’s Benefits Mapping and Analysis Program^43^—Community Edition (BenMAP-CE) Version 1.5.8.23 to compare the attributable counts with those obtained from the *attrdl* function. Details on the methods used for BenMAP comparison are reported in Supplemental Material https://links.lww.com/EE/A437.

## Results

### Overview

**Figure 1.**
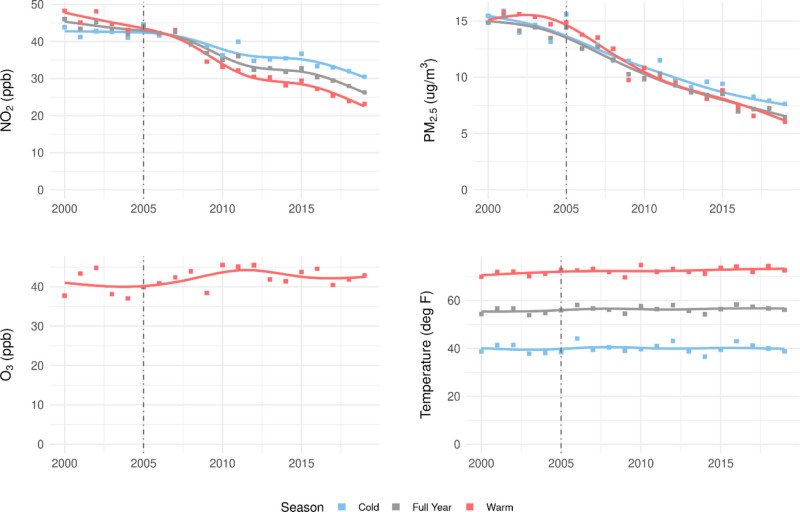
Annual trend in mean daily pollution concentrations and temperature.

**Figure 2.**
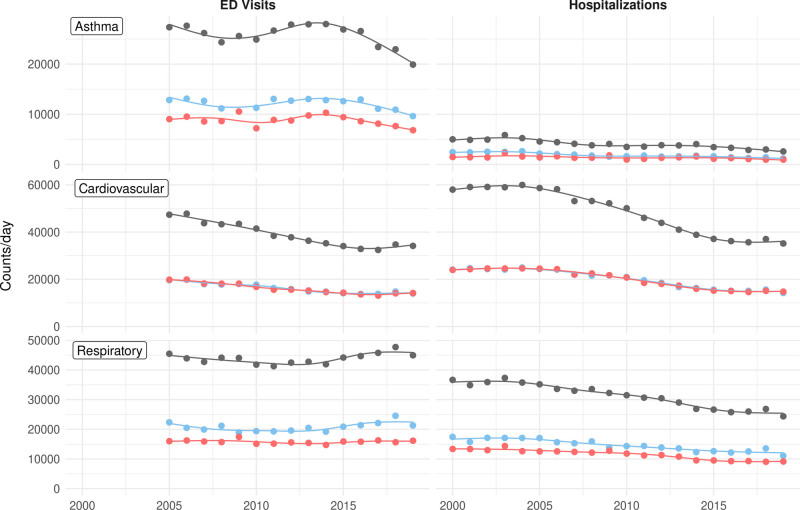
Annual outcome counts by season.

**Figure 3.**
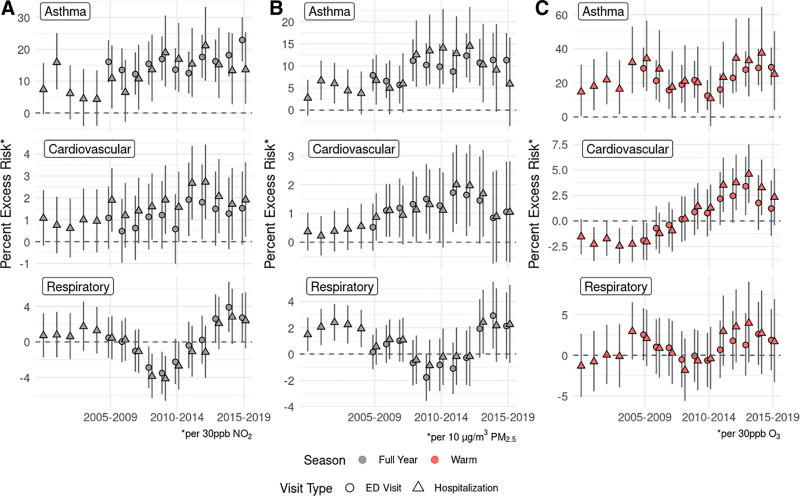
Trends in 5-year moving time window PERs (95% CIs) by outcome for (A) full-year NO_2_, (B) full-year PM_2.5_, and (C) warm season ozone.

**Figure 4.**
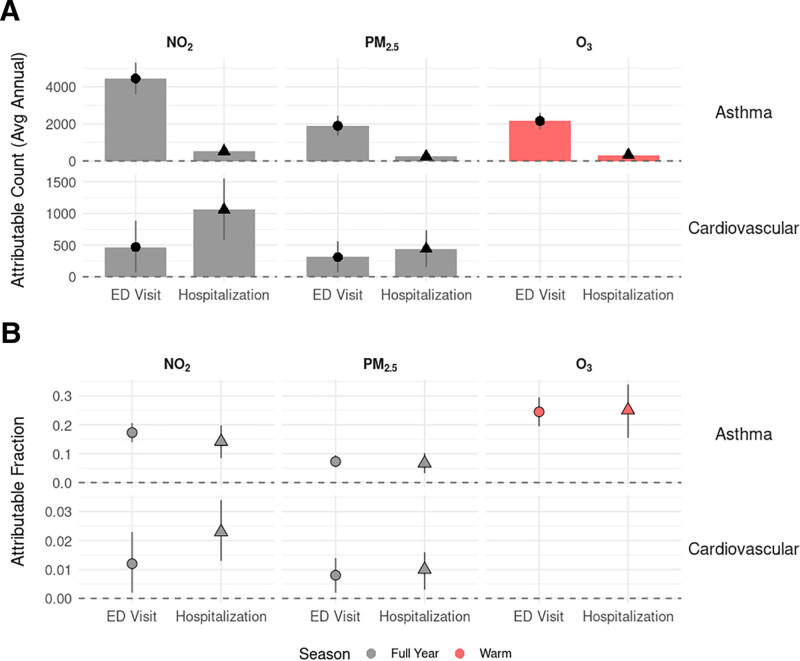
(A) Annual average pollution-attributable ED visits and hospitalizations and (B) pollution-attributable fractions by outcome and pollutant with 95% CIs (2005–2019).

Of all three pollutants evaluated in this study, PM_2.5_ average concentrations declined most rapidly over time (Figure 1). NO_2_ concentrations also declined, while warm-season ozone concentrations remained unchanged. Trends in the health outcomes studied are displayed in Figure 2, and descriptive information on the health burden is summarized in Table 1. From 2005 to 2019, youth asthma and adult cardiovascular ED visits decreased by about 25%, while changes in older adult respiratory ED visits were much less. Hospitalizations also decreased from 2000 to 2019 for all three outcomes. Most pollutant–outcome pairs did not show a monotonic trend in PER over time, and confidence bands widened in the later years for pollutants with declining concentrations (Figure 3). Across all pollutants and outcomes studied, NO_2_ had the largest impacts on the annual average attributable burden (Figure 4). NO_2_- and PM_2.5_-attributable health impacts declined over the 15-year period, with the largest declines shown in PM_2.5_-attributable hospitalizations for all three outcomes. Ozone-attributable burden was mostly unchanged over time, consistent with ozone’s unchanged average concentrations. Seasonal analyses for NO_2_ and PM_2.5_ showed that these pollutants had impacts on asthma in both seasons, but on older adult respiratory morbidity in the warm season and on cardiovascular morbidity in the cold season. The full seasonal results are included in Figures S3 and S4 https://links.lww.com/EE/A437. The cumulative PERs for each pollutant-outcome pair for 2005–2019 are shown in Table 2. Gray-shaded values in Table 2 were not considered in the attributable count and fraction calculations.

**Table 1.** Annual average number of hospitalizations and ED visits by outcome and season

Outcome	Season	Hospitalizations (2000–2019)		ED visits (2005–2019)	
		Number	% from ED	Number	% admitted
Youth asthma (ages 5–17)	Warm	1,369		8,789	
	Cold	1,858		12,034	
	Full year	4,042	91%	25,765	18%
Adult cardiovascular (ages 40+ years)	Warm	20,125		16,091	
	Cold	20,188		16,215	
	Full year	48,587	82%	38,894	77%
Older adult respiratory (ages 65+)	Warm	11,501		15,805	
	Cold	14,643		20,745	
	Full year	31,332	93%	43,884	71%

**Table 2.** Cumulative percent excess risks (PERs) and associated 95% confidence intervals (95% CIs) by outcome and pollutant (2005–2019)

Outcome	Season	Hospitalizations		ED visits	
PM_2.5_		PER	95% CI	PER	95% CI
Youth asthma (ages 5–17)	Full year	5.3	(2.5, 8.3)	8.7	(6.1,11.3)
Adult cardiovascular (ages 40+ years)	Full year	0.6	(0.2, 1.1)	0.8	(0.2, 1.4)
Older adult respiratory (ages 65+)	Full year	1.3	(0.4, 2.1)	0.4	(−0.6, 1.4)
NO_2_					
Youth asthma (ages 5–17)	Full year	12.3	(7.6, 17.3)	17.7	(13.9, 21.6)
Adult cardiovascular (ages 40+ years)	Full year	1.7	(1.0, 2.5)	1.0	(0.1, 1.9)
Older adult respiratory (ages 65+)	Full year	0.1	(−1.2, 1.4)	0.2	(−1.2, 1.7)
Ozone					
Youth asthma (ages 5–17)	Warm	19.1	(10.6, 28.2)	24.0	(18.1, 30.2)
Adult cardiovascular (ages 40+ years)	Warm	−0.5	(−1.5, 0.5)	−0.2	(−1.5, 1.1)
Older adult respiratory (ages 65+)	Warm	0.7	(−1.3, 2.7)	1.4	(−0.5, 3.4)

Percent excess risk (PER) calculated per 30 ppb ozone and NO_2_ and 10 µg/m^3^ PM_2.5_. Cells shaded in gray are models that were excluded from the attributable count calculations.

### Pollution trends

Average daily citywide concentrations of PM_2.5_ declined from about 15 µg/m^3^ in both seasons in 2005 to about 6 µg/m^3^ in 2019 in the warm season and 7.6 µg/m^3^ in the cold season, or about 60% and 50% reductions, respectively. From 2005 to 2019, average daily citywide concentrations of NO_2_ pollution declined from about 44 ppb in both seasons to about 23 ppb in the warm season and 30 ppb in the cold season, or about 50% and 30% reduction, respectively (Figure 1). Ozone’s average concentrations were mostly unchanged over time. Trends in the correlation between pollutant pairs and temperature were published in a previous mortality study (Goldberg et al.). To briefly summarize the temporal correlation changes identified, we found correlations between NO_2_ and PM_2.5_ in the 5-year windows were moderate and somewhat less in more recent years, and in cold months, the correlations were more stable over time. NO_2_’s correlation with ozone in warm months declined, as did NO_2_’s correlations with both temperature terms (full year). The temperature and NO_2_ correlation changed from weakly positive to negative, likely reflecting the larger contribution from building fossil fuel burning compared to traffic sources in more recent years. Correlation coefficients across the 2005–2019 period are reported in Table S1 https://links.lww.com/EE/A437.

### Percent excess risk trends

PM_2.5_-PERs were consistently positive for youth asthma and adult CVD outcomes, with some fluctuation over time (Figure 3). For the respiratory outcomes, some 5-year periods had no association with PM_2.5_. While there appears to be an increase in PM_2.5_-PERs for the cardiovascular outcomes, the later time periods have very wide confidence intervals. The confidence intervals of the risk estimates widened over time for all outcomes as the PM_2.5_ concentrations declined, resulting in greater uncertainty in estimating risk at the 5-year intervals. NO_2_-PERs for youth asthma and CVD outcomes were consistently positive over time. Five-year NO_2_-PERs for the older adult respiratory outcomes dipped negative starting with the PER for the 2007–2011 period, which coincides with the seasonal transition period for both NO_2_ and PM_2.5_ that occurred around 2005 for NO_2_ and 2008 for PM_2.5_. Ozone PERs fluctuated for the youth asthma outcomes but were consistently positive over time. Ozone PERs for the CVD outcomes were mostly null or negative until the 2011–2015 time period, after which they remained positive. We examined the consecutively negative PERs with CVD hospitalizations in the earlier years for both warm-season ozone and NO_2_ and determined that the negative PERs were induced by the adjustment for the same-day temperature (see Supplemental Material and Figures S2A and S13B https://links.lww.com/EE/A437). Nevertheless, we adhered to the main model to conservatively estimate the pollution impacts.

### Pollution-attributable burden

Figure 4 shows the annual average (2005–2019) pollution-attributable ED visits and hospitalizations and respective pollution-attributable fractions. Generally, NO_2_ models produced the largest numbers of pollution-attributable ED visits and hospitalizations for all outcomes except for a few season-specific results (see Figure S3 https://links.lww.com/EE/A437). We estimated about 4500 youth asthma ED visits and 500 NO_2_-attributable hospitalizations (representing about 17% and about 14% of all youth asthma ED visits and hospitalizations, respectively), and 1000 adult CVD NO_2_-attributable hospitalizations and 500 ED visits on average annually (about 2%–4% of the total). PM_2.5_-attributable ED visits were lower, with about 2000 youth asthma ED visits and 300 hospitalizations (about 7% each), and about 300–450 (about 1%) each for adult CVD ED visits and hospitalizations attributable to PM_2.5_ on average annually. As the pollution-attributable burden depends on both total morbidity burden of the outcome and strength of association, the strong cold season-specific associations with CVD outcomes drove the full-year results. For pollution-attributable ED visits and hospitalizations, ozone had a similar impact to PM_2.5_-with more than 2000 ozone-attributable youth asthma ED visits and 320 hospitalizations on average annually—but with a much larger attributable fraction (about 25% vs. 7%–8%) due to generally lower asthma morbidity in the warm season. PM_2.5_-attributable fractions were lower than those for the other pollutants for all outcomes in all seasons except warm-season older adult respiratory hospitalizations (see Figure S4 https://links.lww.com/EE/A437).

### Trends in pollution-attributable burden

**Figure 5.**
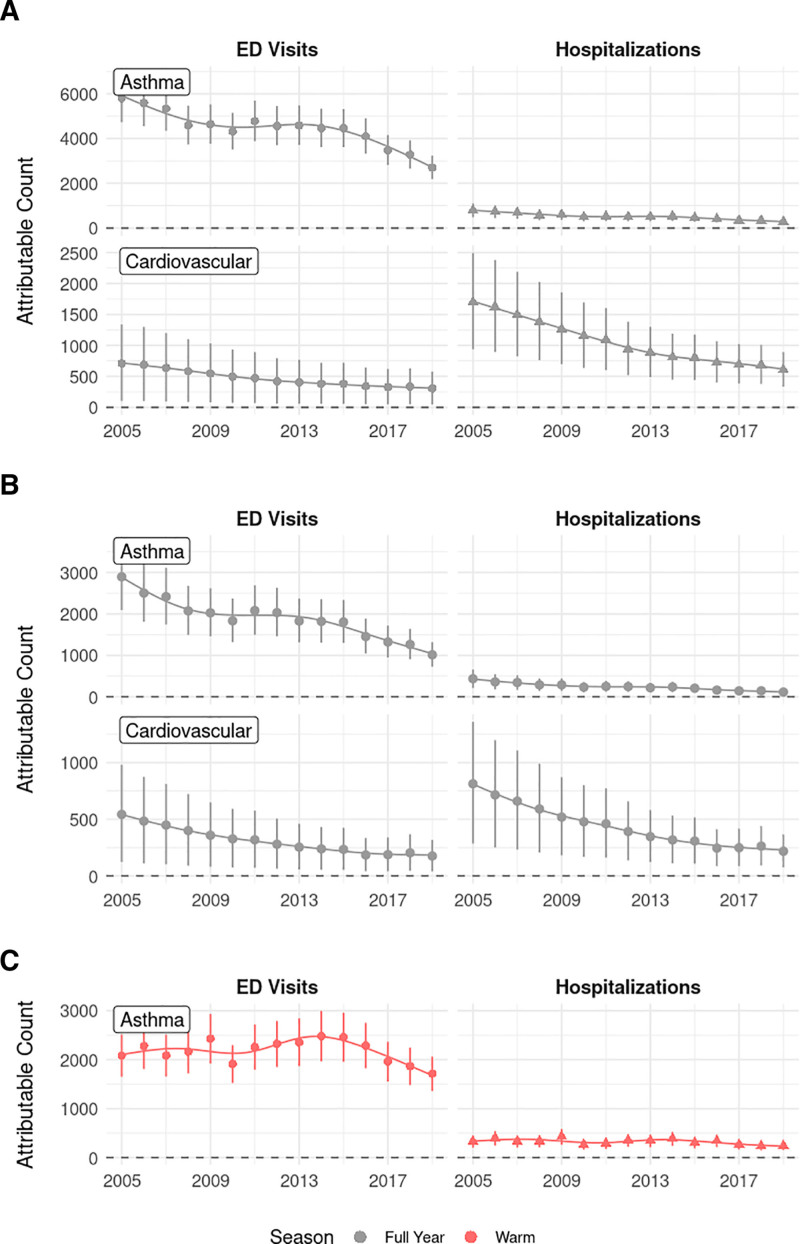
Trends in pollution-attributable ED visits and hospitalizations for (A) NO_2_, (B) PM_2.5_, and (C) ozone.

Trends in the pollution-attributable burden are shown in Figure 5 (and in Figure S5 https://links.lww.com/EE/A437 for season-specific results). Across all outcomes associated with PM_2.5_ for 2005–2019, the attributable counts declined 50%–75% from 2005 to 2019. The largest declines were for youth asthma ED visits and CVD hospitalizations: we estimated about 3000 PM_2.5_-attributable youth asthma ED visits and 800 adult CVD hospitalizations in 2005, compared with about 1000 and 200 in 2019 for each of these outcomes, respectively. The NO_2_-attributable health burden declined 40%–70% depending on the outcome and season, with the largest declines for youth asthma ED visits (from about 6,000 in 2005 to about 2,500 in 2019) and adult CVD hospitalizations (from about 2,000 in 2005 to about 650 in 2019). The ozone-attributable health burden was mostly stable over time, with very small declines in the post-2015 period from changes in total morbidity burden as warm season average ozone concentrations remained unchanged over the study period.

### Sensitivity analysis

Results from our sensitivity analysis are included in the Supplemental Material https://links.lww.com/EE/A437. Sensitivity analyses confirmed the lag structure for each outcome. Risk estimates were similar when employing different degrees of freedom per year for a natural spline of study days to adjust for seasonal trend. Not adjusting for the same-day temperature generally greatly increased PERs for all pollutants for CVD and respiratory ED visits and hospitalizations. Adjustment for co-pollutants showed some attenuation of estimates, but generally, both pollutants remained positively associated with the outcome. The aforementioned results can be found in Figures S6–S11 https://links.lww.com/EE/A437. The asthma warm-season model sensitivity analysis is included in Figure S12A and B https://links.lww.com/EE/A437. Wider confidence intervals were found for the June–August asthma model due to lower counts during the peak of summer, compared with asthma warm-season models including either May or September, or both of these months. The asthma models that included May increased NO_2_ and ozone risk, but decreased PM_2.5_ risk. Trends in attributable burden using the 5-year moving time window PERs showed smaller declines and fluctuations over time (see Figure S15 https://links.lww.com/EE/A437). An average of the attributable counts from the 5-year moving time window PERs produced an overall annual average similar to that calculated from the 2005–2019 model. Comparing attributable counts using the *attrdl* function versus using spatially resolved data in BENMap gave very similar attributable counts, with some pollutant-outcome pairs producing the exact same numbers from both methods, and all producing point estimates that are well within the calculated uncertainties of each method. A side-by-side comparison of these numbers is shown in Tables S3–S5 https://links.lww.com/EE/A437.

## Discussion

Our results support the conclusion that NO_2_ and PM_2.5_ pollution declines in NYC over the last few decades have resulted in fewer pollution-attributable cardiovascular and respiratory exacerbations, resulting in ED visits and/or hospitalizations. With NO_2_ models producing larger attributable counts than PM_2.5_ models, NO_2_ has increased in relative importance in estimating current pollution-attributable morbidity burden. Examining trends in PER for most of the pollutant–outcome pairs showed limited fluctuations over time, with risk estimates becoming less precise as concentrations declined, which is similar to our findings from mortality models.^14^ This makes it harder to conclude that there are distinct increases or decreases in risk over time.

We found a similar pattern of PM_2.5_ associations with CVD morbidity to those published in two previous NYC studies,^24,34^ a study of NYS with regional estimates,^44^ and a nationwide multicity study,^45^ with PM_2.5_ CVD morbidity associations stronger in the cold season than warm season, and same-day effects larger than lagged effects. Despite differences between this study and the previous studies in time periods, health outcome data sources, and diagnoses codes used, the similarity in overall PM_2.5_ PERs shows that the estimate of risk remains similar (about 1%–2% per 10 μg/m^3^) and can be estimated with greater precision when modeled over a long time period.

For asthma–air pollution impacts, our results are consistent with the known impacts of air pollution on asthma exacerbation.^30^ Comparing our results with two previous NYC studies,^25,46^ we found larger risk estimates due to the inclusion of multiple lag days and positive cold-season NO_2_ associations. Consistent with Ito et al., we also found that NO_2_ was a robust predictor of asthma ED visits in co-pollutant models and produced the largest effect estimates compared with the other pollutants studied. A case study in Atlanta using a developmental version of BenMAP-CE to quantify multipollutant health benefits similarly found NO_2_ to be important when estimating pollution-attributable asthma ED visit reductions.^47^

A series of studies conducted by researchers from New York State investigated the relationships between PM_2.5_ and its chemical constituents and CVD hospitalizations,^15,16^ ED visits and hospitalizations for respiratory infections,^17^ and asthma and COPD ED visits and hospitalizations^18^ in urban centers in New York State, including three locations in NYC. Their study periods overlapped ours; however, because their study design utilized both temporal and spatial exposure contrasts (i.e., case-crossover with cases chosen based on the distance from residential address to PM_2.5_ monitors) and they focused on PM_2.5_ (no gaseous pollutants considered), comparing their results to our findings is not straightforward. However, their PM_2.5_ risk estimates either did not decline or increased for the later years for CVD hospitalizations,^15^ asthma and COPD,^18^ and respiratory infection ED visits and hospitalizations.^17^ These findings are consistent with our PM_2.5_ findings, but we also found similar patterns for ozone and warm-season NO_2_.

The pattern of fluctuations in PERs over the study period we observed for cardiovascular and respiratory hospitalizations (Figure 3) is similar to those reported by Chen et al. for PM_2.5_ and circulatory (increasing) and respiratory (decreased approximately between 2007 and 2012, followed by an increase) hospitalizations among Medicare beneficiaries for 968 US counties.^13^ However, in our analysis, a similar pattern was also observed for NO_2_ and ozone, and therefore, this pattern of fluctuations in PERs is unlikely to be limited to the factors related to PM_2.5_ (e.g., change in its chemical constituents).

We found that the NO_2_-attributable impact on both asthma and CVD hospitalizations was more than double the PM_2.5_-attributable impact, and the decline in NO_2_-attributable impact was less than that for PM_2.5_. Thus, while reductions in PM_2.5_-attributable counts achieved substantial health benefits, NO_2_ is becoming a more important predictor of pollution impact, aligning with the updated causal determination for NO_2_ in the EPA’s 2016 Integrated Science Assessments to “causal” for short-term respiratory effects and “suggestive of” causal for short-term cardiovascular effects.^41^ Similar to findings presented here, in our mortality study, we also found stronger NO_2_ impacts from pollution over the last 2 decades, demonstrating policy-relevant consistency in NO_2_ impacts across health outcomes.^14^ Reporting only the PM_2.5-_attributable burden may underestimate the pollution-attributable impacts from local combustion sources that contribute to both PM_2.5_ and NO_2_ concentrations in urban environments like NYC. Comparison of attributable burden can be useful for analyzing the changing regulatory landscape, including expected air pollution reduction co-benefits from local and state climate policy goals.^4,48,49^ For example, in NYC, almost half of the oxides of nitrogen (NO_*x*_) emissions are from the building sector,^50^ making electrification of building heating systems both a key climate mitigation goal and a potential driver of averted health impacts from NO_2_. Similarly, 13% of NO_*x*_ emissions in NYC are produced by heavy-duty vehicles,^50^ making the health impacts of NO_2_ an important and significant co-benefit of the 2027 emissions standard for heavy-duty commercial vehicles. NO_2_ is a precursor of nitrate, which is a significant fraction of PM_2.5_ in NYC, especially in winter, and also contributes to the formation of summertime ozone, so reducing NO_2_ sources can also reduce health impacts attributable to these pollutants.^20^ Reducing NO_2_ concentrations can also improve baseline health long-term.^51,52^ An analysis of children’s asthma incidence from the Global Burden of Disease study found that 16% of incident asthma was attributable to NO_2_ in 2019, down from 19% in 2000, but still rising in poorer urban regions of the world.^51^

The findings of this study not only support efforts to improve air quality but also underscore the importance of improving overall population health. Efforts to improve cardiovascular health in the NYC population have had some success and include smoking cessation efforts and advancements in medical care.^53^ Similarly, multifactorial approaches to reducing asthma burden are needed, including improvements in medication development and adherence, and reductions in exposure to environmental allergens (e.g., mold, pests, dust) that contribute to poor indoor air quality.^30,54^ The interplay of diagnoses along the continuum of disease and the importance of primary prevention is paramount. For example, children with asthma exacerbations visiting the ED today may become future adult cardiovascular disease patients with an increased risk of premature death.^55,56^ Tackling systemic issues to improve access to quality healthcare,^57^ provide quality housing,^58^ and implement community interventions^59^ can meaningfully impact morbidity burden and, in turn, can reduce air pollution-attributable exacerbations, their associated healthcare costs, and premature death.

Strengths of this study include the long study period and the large volume of morbidity counts in a relatively small geographic area that experienced a substantial decline in air pollution levels, allowing us to conduct time-stratified analyses, as well as our direct calculation of attributable burden for multiple health outcomes. Limitations include the use of citywide concentrations, which limited our ability to determine spatial differences in exposure. However, NYC is the most densely populated major city in the United States with a relatively small area, so spatial exposure misclassification error is expected to be small for these mostly spatially uniform pollutants. In addition, comparison with BenMAP-CE output that uses spatially resolved pollution data showed minimal differences from our findings. The fluctuations in attributable burden and smaller downward trend we found when calculated from the 5-year moving time window PERs rather than the 2005–2019 PER limit our ability to definitively conclude the magnitude of decline and highlight that our main results do not completely take into account all factors that may play a role in air pollution health impacts over time, such as the changing relationships of pollutants with temperature observed over time. This analysis was also limited to the overall city population and did not attempt to identify at-risk subgroups. We are currently conducting analyses stratified by race and ethnicity, and poverty, to determine the populations most affected.

## Conclusion

We observed substantial reductions in estimated morbidity counts attributed to PM_2.5_ and NO_2_ during the study period, providing evidence of the benefits of emission regulation efforts. NO_2_-attributable morbidity burden was larger than PM_2.5_-attributable morbidity burden, and the decline in NO_2_ concentrations has been slower. Future HIAs of energy and air quality interventions should incorporate NO_2_ to best capture the effects of local air pollution sources (i.e., traffic and buildings). The short-term exacerbation of pre-existing conditions by air pollution can also be reduced by improving baseline morbidity through public health programs.

## Conflict of interest statement

The authors declare that they have no conflicts of interest with regard to the content of this report.

## Data availability

Data are available from the New York State Department of Health (NYSDOH) by application and a signed data user agreement. Health outcome data used in our analysis were extracted from the Statewide Planning and Research Cooperative System (SPARCS). Because of slight variations between datasets, results might differ from those calculated using a different extract of the NYSDOH SPARCS. Analyses include data from SPARCS 2000–2019, which were cut by NYSDOH on the following dates: 2000–2007 (July 2014); 2008–2012 (July 2017); 2013–2015 (January 2018); 2016–2018 (July 2022); 2019 (January 2023). Internal analyses by the New York City Department of Health and Mental Hygiene were conducted in July 2022 (2000–2018 data) and May 2023 (2019 data).

## Disclaimer

This publication was produced from raw data purchased from or provided by the New York State Department of Health. However, the calculations, metrics, conclusions derived, and views expressed herein are those of the authors and do not reflect the conclusions or views of NYSDOH. NYSDOH, its employees, officers, and agents make no representation, warranty, or guarantee as to the accuracy, completeness, currency, or suitability of the information provided here.

## Supplementary Material


