# Supplementary material for: Changes in respiratory and cardiovascular morbidity air pollution risk and attributable burden in New York City
Source: Environ Epidemiol. 2026 Jul 21;10(4):e497. doi: 10.1097/EE9.0000000000000497 (PMC13391141; doi:10.1097/EE9.0000000000000497)
Supplement: Supplementary file 1 [file ee9-10-e497-s001.pdf]

Supplemental Information for Changes in respiratory and cardiovascular morbidity air pollution risk and attributable burden in New York City Ariel Spira-Cohen, Rebecca Goldberg, Sarah Johnson, Masha Pitiranggon, Kazuhiko Ito

Table of Contents:

Text and Table/Figure	Pages
Development of the daily citywide average concentrations of $PM_{2.5}$, O_3, and NO_2. (text and Figure S1)	3-4
Table S1. Mean concentrations, standard deviation and Pearson correlation matrix for daily exposure variables by season and for full year (2005-2019).	5
Table S2: Annual average pollution attributable counts and fractions by pollutant, outcome and season with 95% Confidence Intervals (2005-2019)	5-6
Season specific results (text)	7
Figure S-2: Trends in 5-year moving time window PERs by outcome for warm and cold season A) NO_2 and B) $PM_{2.5}$.	8
Figure S-3: Seasonal annual average pollution-attributable ED visits and hospitalizations and 95% CI's by pollutant and outcome	9
Figure S-4: Seasonal pollution attributable fractions with 95% CI's by pollutant and outcome.	9
Figure S-5: Trends in pollution-attributable ED visits and hospitalizations for (A) NO_2 (B) $PM_{2.5}$	10
Figure S-6: Model sensitivity of percent excess risks for asthma ED visits, 2005-2019.	11
Figure S-7: Model sensitivity of percent excess risks for asthma hospitalizations, 2005-2019.	12
Figure S-8: Model sensitivity of percent excess risks for cardiovascular ED visits, 2005-2019.	13
Figure S-9: Model sensitivity of percent excess risks for cardiovascular hospitalizations, 2005-2019.	14
Figure S-10: Model sensitivity of percent excess risks for respiratory ED visits, 2005-2019.	15
Figure S-11: Model sensitivity of percent excess risks for respiratory hospitalizations, 2005-2019.	16
Asthma warm season model sensitivity analysis (text and Figure S-12A and S-12B	17
Analysis of NO_2 and O_3 with CVD hospitalizations in the warm months (text and Figure S-13A and S-13B)	18-19
Figure S-14. Trend in pairwise correlation among air pollutants and temperature variables in the 5-year moving time windows.	20

BENMAP versus attrdl comparison (text and Tables S-3, S-4, S-5)	21-23
Trends in pollution attributable burden using 5-year moving time windows (text and Figure S-15)	23-24
Citations for Supplemental Material	25

Supplemental Information for Changes in respiratory and cardiovascular morbidity air pollution risk and attributable burden in New York City Ariel Spira-Cohen, Rebecca Goldberg, Sarah Johnson, Masha Pitiranggon, Kazuhiko Ito

Development of the daily citywide average concentrations of $PM_{2.5}$, O_3, and NO_2.

We retrieved $PM_{2.5}$, NO_2, and O_3 data from U.S. Environmental Protection Agency's (EPA's) Air Quality System [1] for the five boroughs in NYC and counties in New Jersey state adjacent to New York City (NYC). We used metrics consistent with the National Ambient Air Quality Standard for each pollutant: NO_2: daily 1-hr max; O_3: daily 8-hr max; and $PM_{2.5}$: 24-hr average. The original citywide average air pollution dataset for this analysis was developed for a longer period, 1990-2019, than the period covered in the current analysis (2000-2019) because the project included analysis for earlier years when the health outcome data (i.e., mortality) were available. The motivation for preparing complete daily time-series air pollution data without missing values was the need to compute the attributable counts of the health outcomes for the entire study period without days with missing data for the three pollutants. A previous study [2] of air pollution and asthma emergency department visits in NYC (1999-2002) used the citywide average values computed from multiple monitors within a 20-mile (32 kilometers) radius from the geographic center of NYC, including the monitors in New Jersey State. The 2007 study reported that the monitor-to-monitor temporal correlations for $PM_{2.5}$, NO_2, and O_3 were high (median correlations ~0.9), with little decay in correlation by their separation distance. Based on this information, we processed data from multiple stations through the steps: (1) map all the monitors with monitoring objectives of "population exposure" in NYC and New Jersey counties adjacent to NYC (Bergen, Passaic, Hudson, Essex, Union, Middlesex, and Monmouth counties); (2) retrieve the daily and site information data, compute temporal correlations of all pairs of the sites and eliminate the sites that were correlated poorly with the NYC sites; (3) compute the distance between each pair of monitors and plot them against the corresponding correlations to ensure the inclusion of these monitors is appropriate; and (4) compute the citywide average that takes into consideration in the difference in mean and variance of individual sites using the method developed by Schwartz. [3]

Figure S-1 shows a map of all the monitors that were included in the computation of daily multi-site average air pollution values. These included 39 monitors (13 of which from New Jersey) for $PM_{2.5}$, 19 monitors (9 of which from New Jersey) for NO_2, and 19 monitors (7 of which from New Jersey) for O_3. The median monitor-to-monitor correlations among these monitors for $PM_{2.5}$, NO_2, and O_3 were 0.90, 0.69, and 0.90, respectively. After computing multi-site averages for the years used for this analysis, 2000 through 2019, there were no missing values for NO_2 and O_3, and 18 missing values for $PM_{2.5}$. Based on the observation that the 1-day lag autocorrelation of $PM_{2.5}$ was relatively high (~0.6), we filled in these 18 missing values using either the previous day's or the following day's values. There were no consecutively missing values longer than two days.

Supplemental Information for Changes in respiratory and cardiovascular morbidity air pollution risk and attributable burden in New York City Ariel Spira-Cohen, Rebecca Goldberg, Sarah Johnson, Masha Pitiranggon, Kazuhiko Ito

Figure S-1. Location of air quality monitors in New York City and New Jersey counties adjacent to New York City.

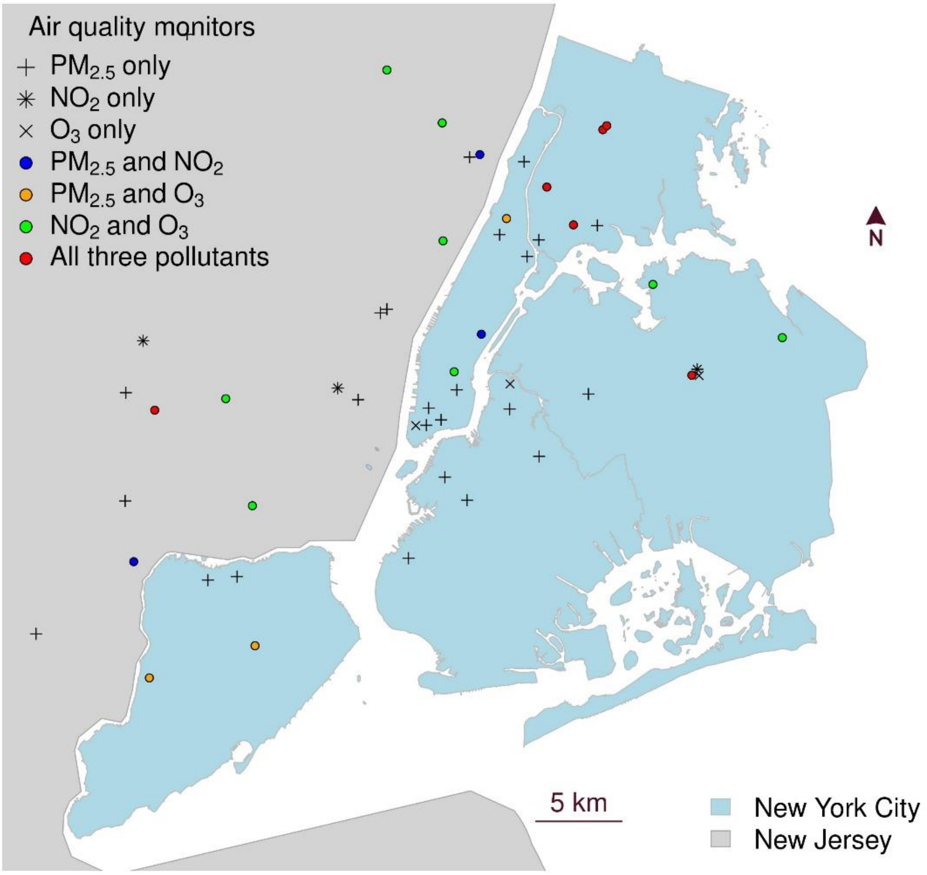

Supplemental Information for Changes in respiratory and cardiovascular morbidity air pollution risk and attributable burden in New York City Ariel Spira-Cohen, Rebecca Goldberg, Sarah Johnson, Masha Pitiranggon, Kazuhiko Ito

Table S1. Mean concentrations, standard deviation and Pearson correlation matrix for daily exposure variables by season and for full year (2005-2019).

	MEAN	STANDARD DEVIATION	$PM_{2.5}$	NO_2	O_3	MEAN TEMP	LAGS 1-3 TEMP AVG
**WARM**							
**$PM_{2.5}$ (μG/M3)**	9.85	6.47		0.59	0.58	0.51	0.27
**NO_2 (PPB)**	32.4	13.1			0.36	0.16	-0.08
**O_3**	42.6	14.4				0.60	0.33
**COLD**							
**$PM_{2.5}$ (μG/M3)**	10.26	6.16		0.64	N/A	0.07	-0.24
**NO_2 (PPB)**	37.0	12.2			N/A	0.18	-0.17
**FULL YEAR**							
**$PM_{2.5}$ (μG/M3)**	9.62	6.13		0.61	N/A	0.12	-0.03
**NO_2 (PPB)**	34.7	13.1			N/A	-0.05	-0.20

Table S2: Annual average pollution attributable counts and fractions by pollutant, outcome and season with 95% Confidence Intervals (2005-2019)

OUTCOME	SEASON	HOSPITALIZATIONS		ED VISITS	
		Fraction	Count	Fraction	Count
**$PM_{2.5}$**					
**YOUTH ASTHMA (AGES 5-17)**	Warm	7.6% (1.0; 14)	97 (13; 180)	6.1% (2.7; 9.6)	535 (235; 839)
	Cold	7.8% (3.3; 12.4)	129 (55; 204)	8.3% (5.9; 10.8)	1,000 (706; 1302)
	Full Year	6.7% (3.3; 10.2)	246 (120; 374)	7.3% (5.3; 9.5)	1,894 (1,363; 2,447)
**ADULT CARDIOVASCULAR (AGES 40+ YEARS)**	Warm	X	X	X	X
	Cold	1.5% (0.5; 2.5)	284 (97; 478)	1.6% (0.7; 2.6)	265 (111; 426)
	Full Year	1.0% (0.3; 1.6)	438 (154; 735)	0.8% (0.2; 1.4)	309 (70; 559)
**OLDER ADULT RESPIRATORY (AGES 65+)**	Warm	3.6% (1.8; 5.3)	393 (199; 575)	1.7% (0.2; 3.2)	275 (35; 502)
	Cold	X	X	X	X

	Full Year	X	X	X	X
**NO_2**					
**YOUTH ASTHMA (AGES 5-17)**	Warm	20% (11.6; 28.2)	258 (149; 362)	18.2% (13.6; 22.8)	1,599 (1,195; 2,003)
	Cold	8.3% (-1.7; 17.6)	137 (-27; 290)	12.4% (7.3; 17.4)	1,490 (882; 2,090)
	Full Year	14.2% (8.5; 19.8)	519 (313; 723)	17.3% (14.0; 20.6)	4,449 (3,611; 5,303)
**ADULT CARDIOVASCULAR (AGES 40+ YEARS)**	Warm	X	X	X	X
	Cold	3.1% (1.2; 5.1)	584 (222; 956)	2.1% (0.3; 4.0)	340 (41; 649)
	Full Year	2.3% (1.3; 3.4)	1,058 (583; 1,552)	1.2% (0.2; 2.3)	469 (69; 884)
**OLDER ADULT RESPIRATORY (AGES 65+)**	Warm	1.7% (-1.0; 4.3)	184 (-106; 463)	1.4% (-0.9; 3.5)	214 (-135; 549)
	Cold	X	X	X	X
	Full Year	X	X	X	X
**O_3**					
**YOUTH ASTHMA (AGES 5-17)**	Warm	25.1% (15.5; 34)	321 (198; 436)	24.5% (19.5; 29.5)	2,155 (1,710; 2,595)
**ADULT CARDIOVASCULAR (AGES 40+ YEARS)**	Warm	X	X	X	X
**OLDER ADULT RESPIRATORY (AGES 65+)**	Warm	X	X	X	X

Season-specific results.

Seasonal models were defined as May-Sept for warm season and Nov-March for cold season. Only the asthma outcomes had positive associations in both seasons for all pollutants. The seasonal PERs from the 5-year moving time window models are shown in Figure S-2. NO_2PERs for youth asthma outcomes were consistently positive in the warm season, but only consistently positive for ED visits (not hospitalizations) in the cold season. Cardiovascular outcomes had positive associations in the cold season and full year, and positive associations for older adult respiratory outcomes were mostly found in the warm season. CVD outcomes showed a seasonal converging of PERs in the more recent 5-year time periods, with an increase in warm season PERs and reduction in cold season PERs.

Annual average attributable count and fraction for the seasonal models for NO_2 and $PM_{2.5}$ are shown in Figures S-3 and S-4, respectively. Despite a much higher warm season PER (2-3 times larger in magnitude), the NO_2 attributable asthma ED visits were similar in both seasons, and a larger fraction of the asthma ED visits were attributable to NO_2 in the warm season than cold season (18-20% versus 8-12%). Cardiovascular outcomes were only associated in the cold season, and we found higher attributable burden from NO_2 than $PM_{2.5}$, with more than 500 NO_2-attributable CVD hospitalizations on average per year. For the older adult respiratory outcomes, about 300 ED visits and 400 hospitalizations were attributable to $PM_{2.5}$ in the warm season, or about 2-4% of the total, and larger than those attributable to NO_2. Trends in the attributable burden showed declines in both seasons for $PM_{2.5}$ and NO_2 attributable outcomes. $PM_{2.5}$-attributable asthma ED visits declined more in the cold season than warm season.

Supplemental Information for Changes in respiratory and cardiovascular morbidity air pollution risk and attributable burden in New York City Ariel Spira-Cohen, Rebecca Goldberg, Sarah Johnson, Masha Pitiranggon, Kazuhiko Ito

Figure S-2: Trends in 5-year moving time window PERs by outcome for warm and cold season A) NO_2 and B) $PM_{2.5}$.

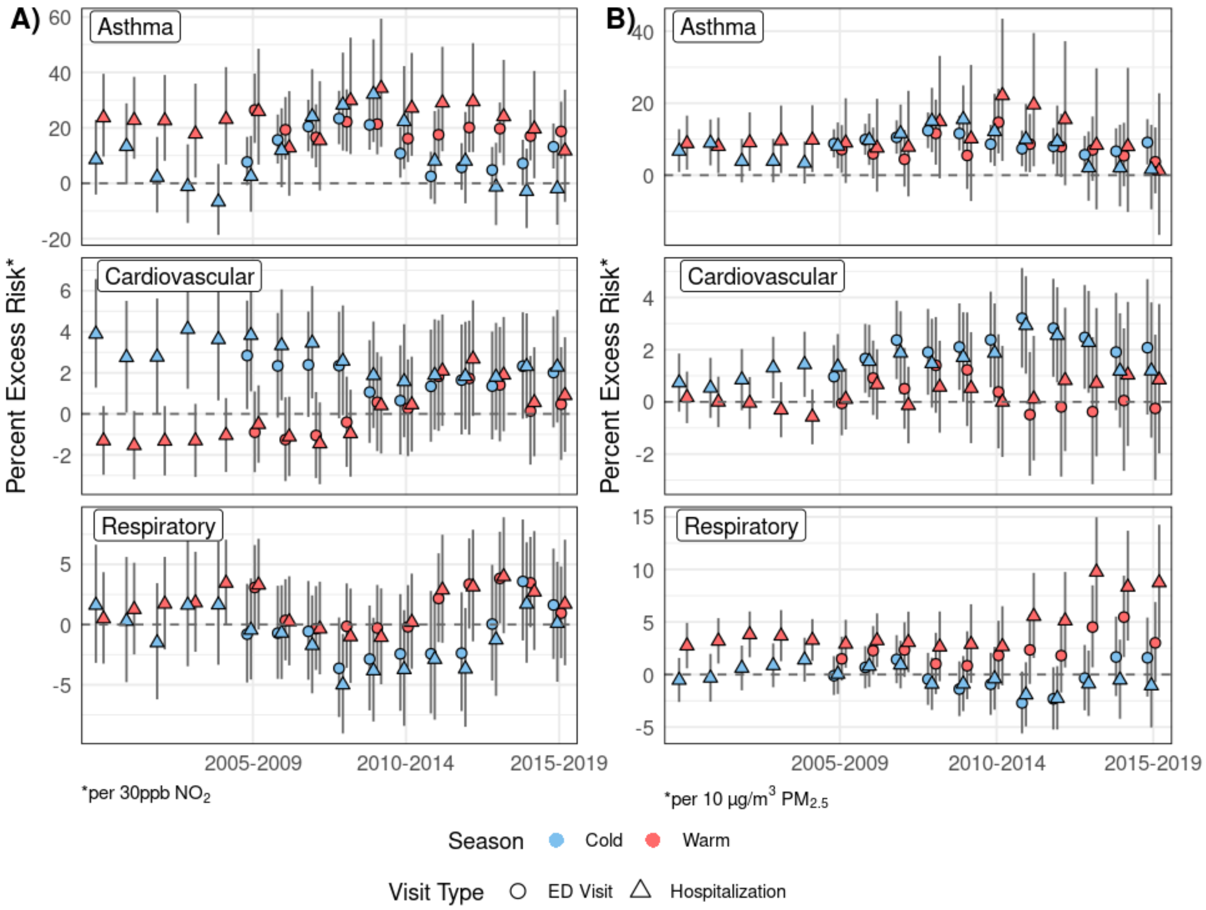

Supplemental Information for Changes in respiratory and cardiovascular morbidity air pollution risk and attributable burden in New York City Ariel Spira-Cohen, Rebecca Goldberg, Sarah Johnson, Masha Pitiranggon, Kazuhiko Ito

Figure S-3: Seasonal annual average pollution-attributable ED visits and hospitalizations and 95% CI's by pollutant and outcome.

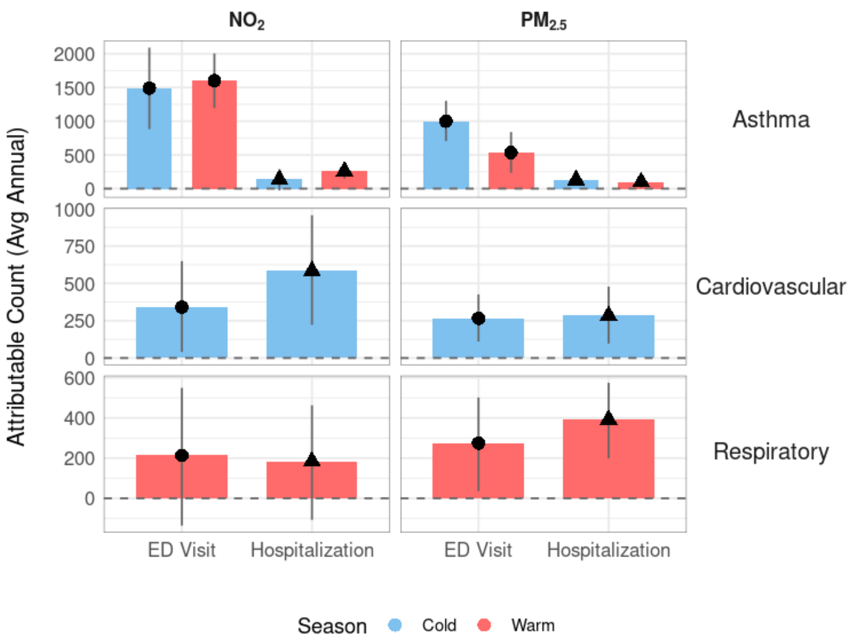

Figure S-4: Seasonal pollution attributable fractions with 95% CI's by pollutant and outcome.

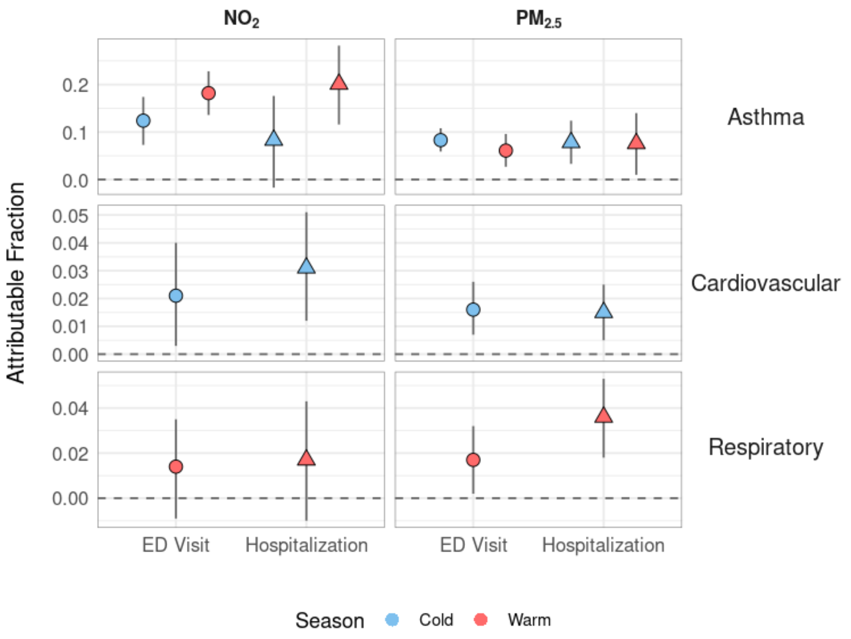

Supplemental Information for Changes in respiratory and cardiovascular morbidity air pollution risk and attributable burden in New York City Ariel Spira-Cohen, Rebecca Goldberg, Sarah Johnson, Masha Pitiranggon, Kazuhiko Ito

Figure S-5: Trends in pollution-attributable ED visits and hospitalizations for (A) NO_2 (B) $PM_{2.5}$

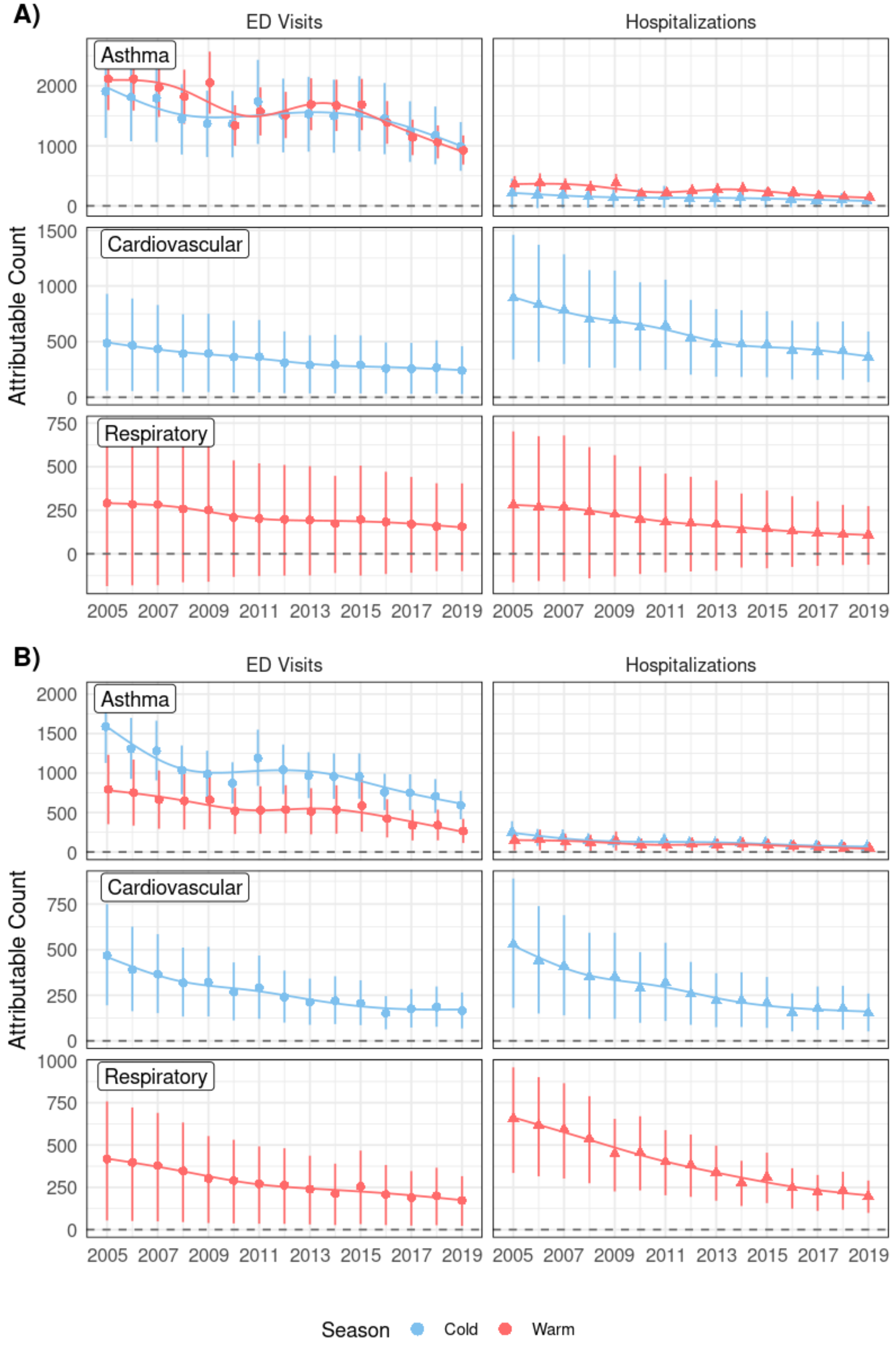

Supplemental Information for Changes in respiratory and cardiovascular morbidity air pollution risk and attributable burden in New York City Ariel Spira-Cohen, Rebecca Goldberg, Sarah Johnson, Masha Pitiranggon, Kazuhiko Ito

Figure S-6: Model sensitivity of percent excess risks for asthma ED visits, 2005-2019.

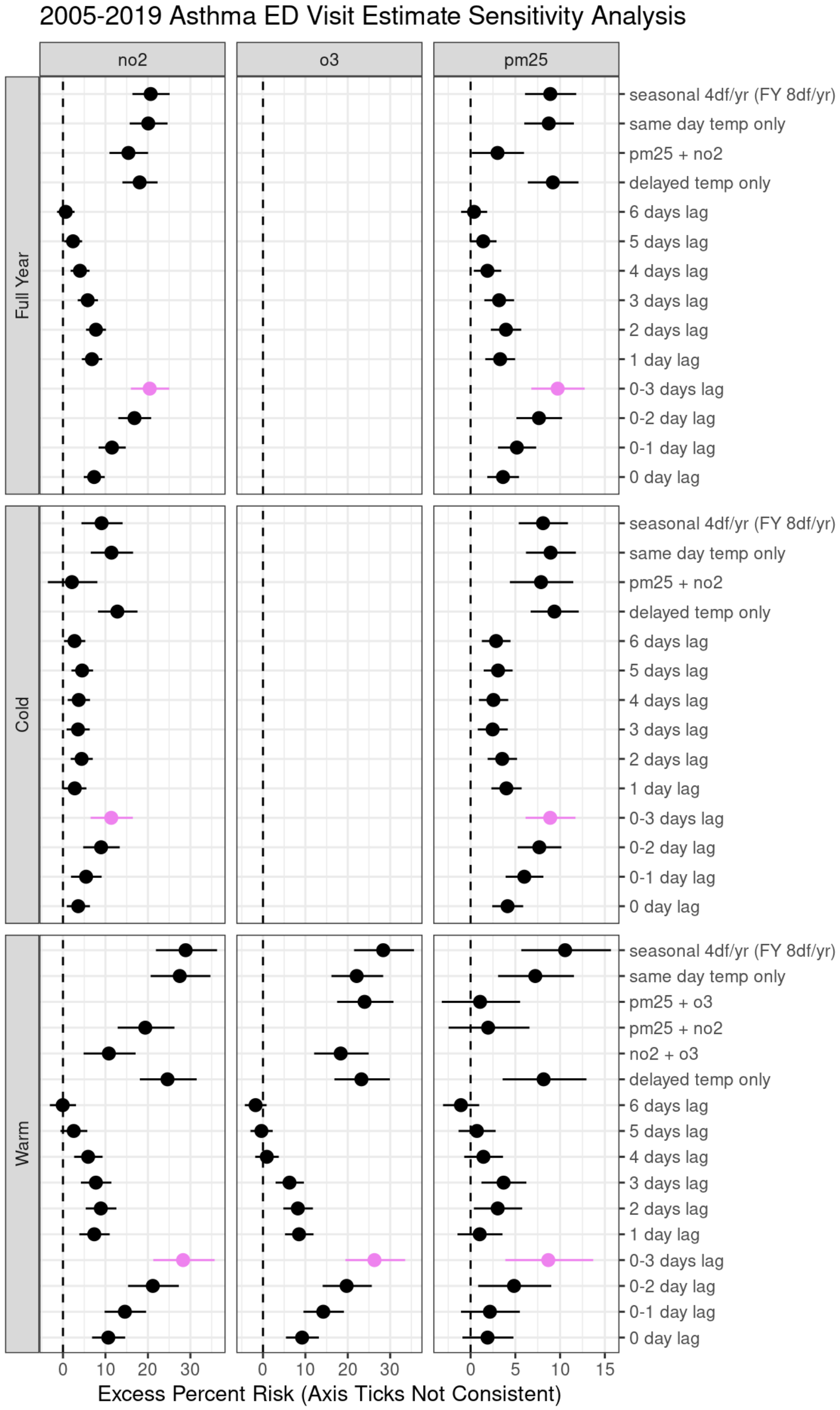

Supplemental Information for Changes in respiratory and cardiovascular morbidity air pollution risk and attributable burden in New York City Ariel Spira-Cohen, Rebecca Goldberg, Sarah Johnson, Masha Pitiranggon, Kazuhiko Ito

Figure S-7: Model sensitivity of percent excess risks for asthma hospitalizations, 2005-2019.

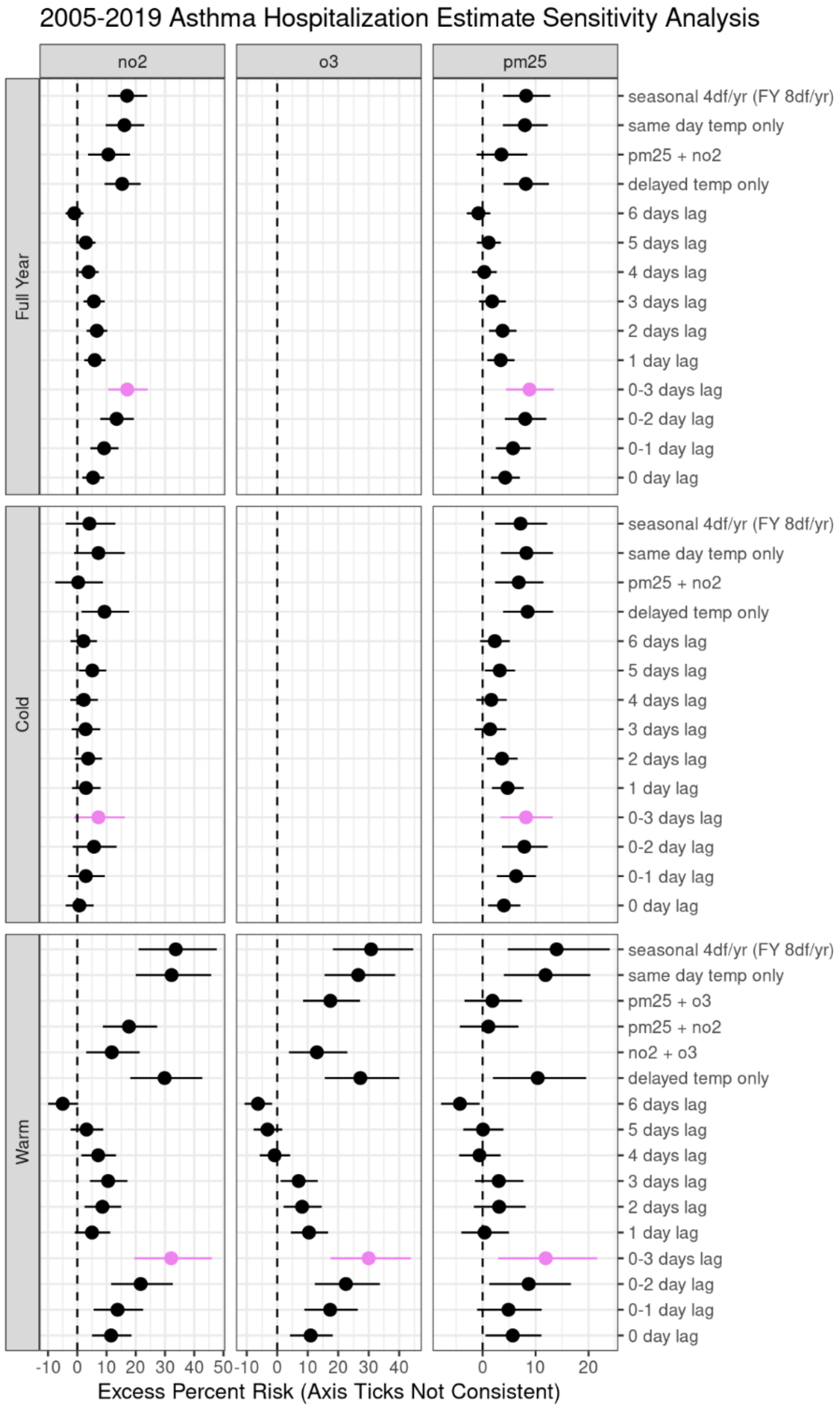

Supplemental Information for Changes in respiratory and cardiovascular morbidity air pollution risk and attributable burden in New York City Ariel Spira-Cohen, Rebecca Goldberg, Sarah Johnson, Masha Pitiranggon, Kazuhiko Ito

Figure S-8: Model sensitivity of percent excess risks for cardiovascular ED visits, 2005-2019.

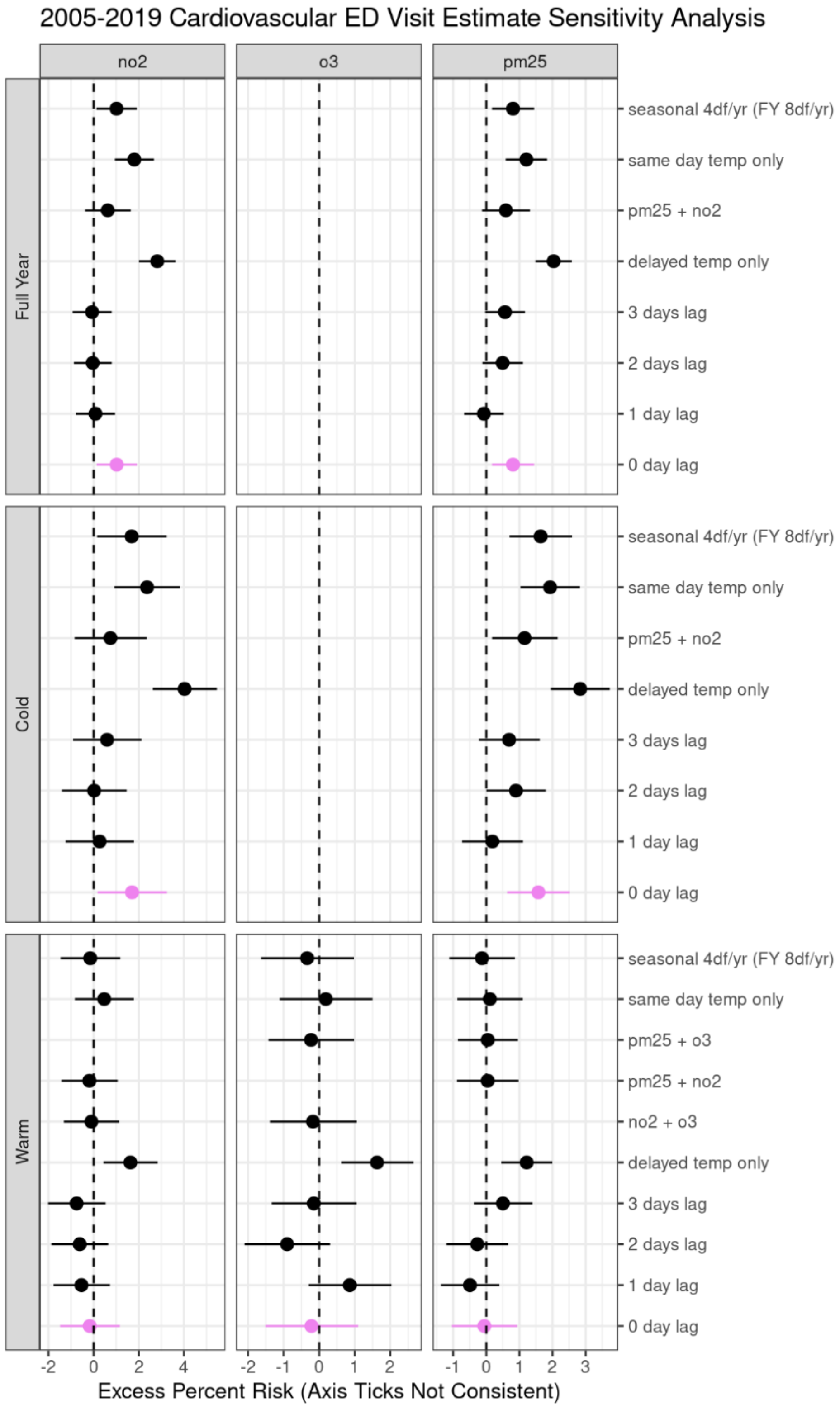

Supplemental Information for Changes in respiratory and cardiovascular morbidity air pollution risk and attributable burden in New York City Ariel Spira-Cohen, Rebecca Goldberg, Sarah Johnson, Masha Pitiranggon, Kazuhiko Ito

Figure S-9: Model sensitivity of percent excess risks for cardiovascular hospitalizations, 2005-2019.

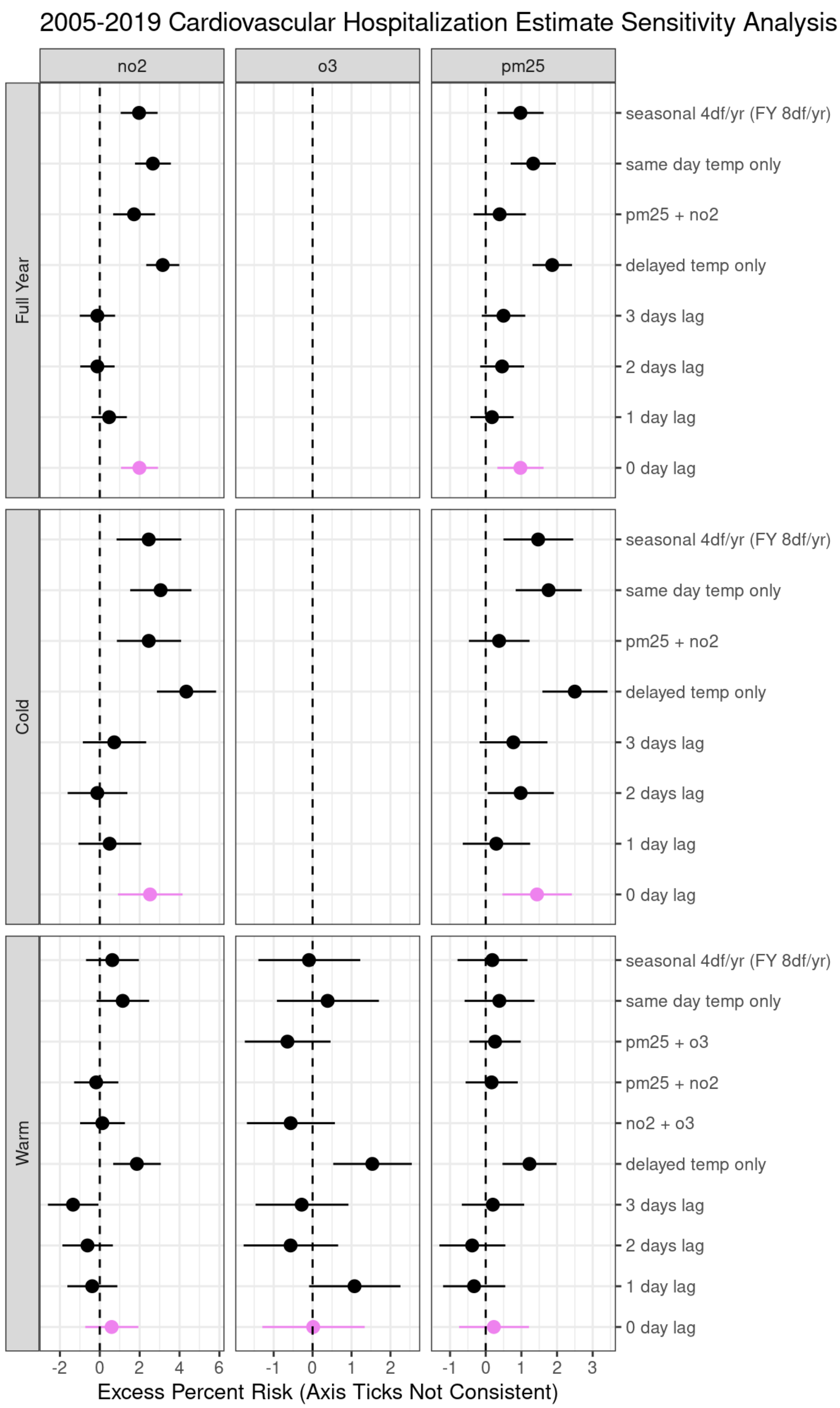

Supplemental Information for Changes in respiratory and cardiovascular morbidity air pollution risk and attributable burden in New York City Ariel Spira-Cohen, Rebecca Goldberg, Sarah Johnson, Masha Pitiranggon, Kazuhiko Ito

Figure S-10: Model sensitivity of percent excess risks for respiratory ED visits, 2005-2019.

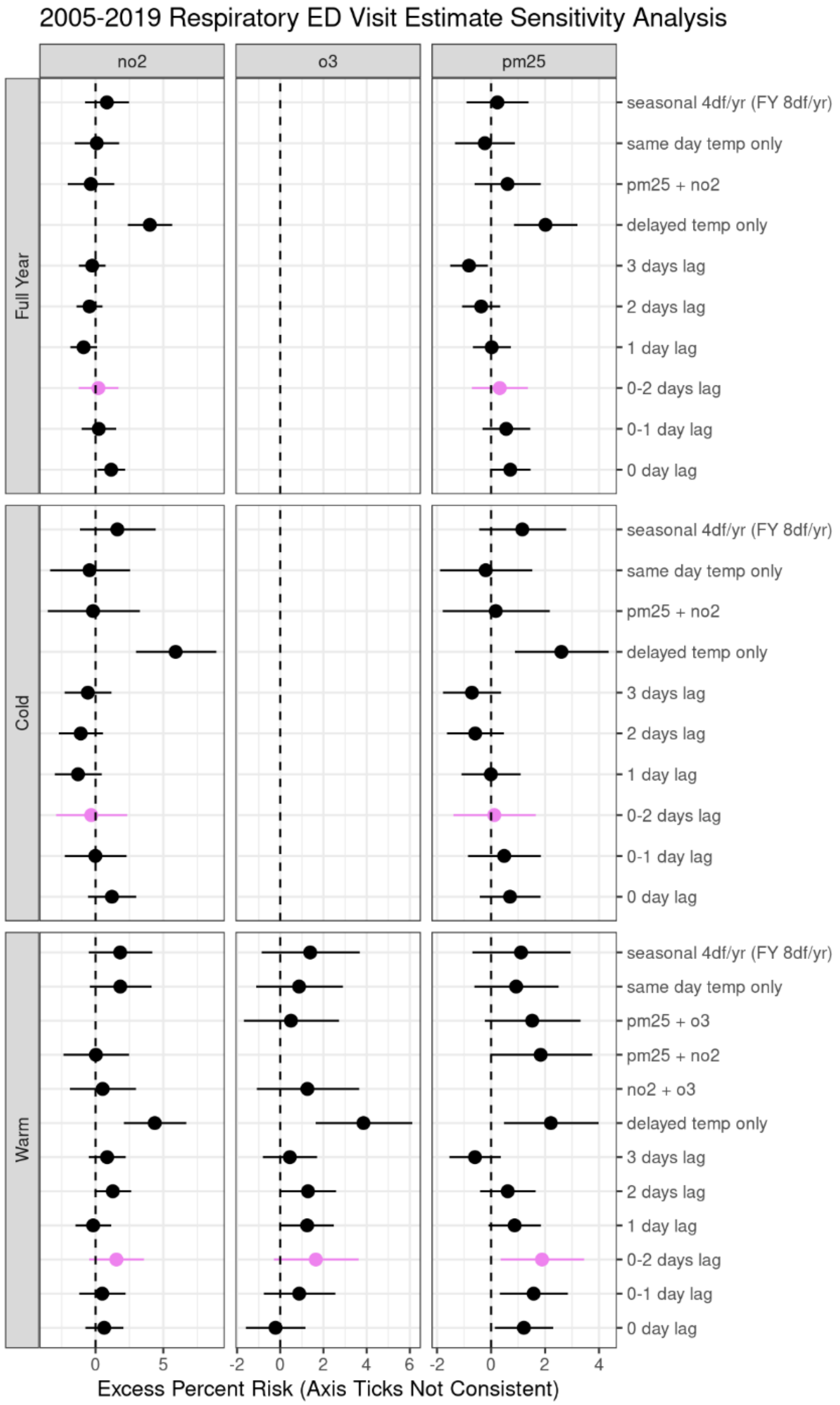

Supplemental Information for Changes in respiratory and cardiovascular morbidity air pollution risk and attributable burden in New York City Ariel Spira-Cohen, Rebecca Goldberg, Sarah Johnson, Masha Pitiranggon, Kazuhiko Ito

Figure S-11: Model sensitivity of percent excess risks for respiratory hospitalizations, 2005-2019.

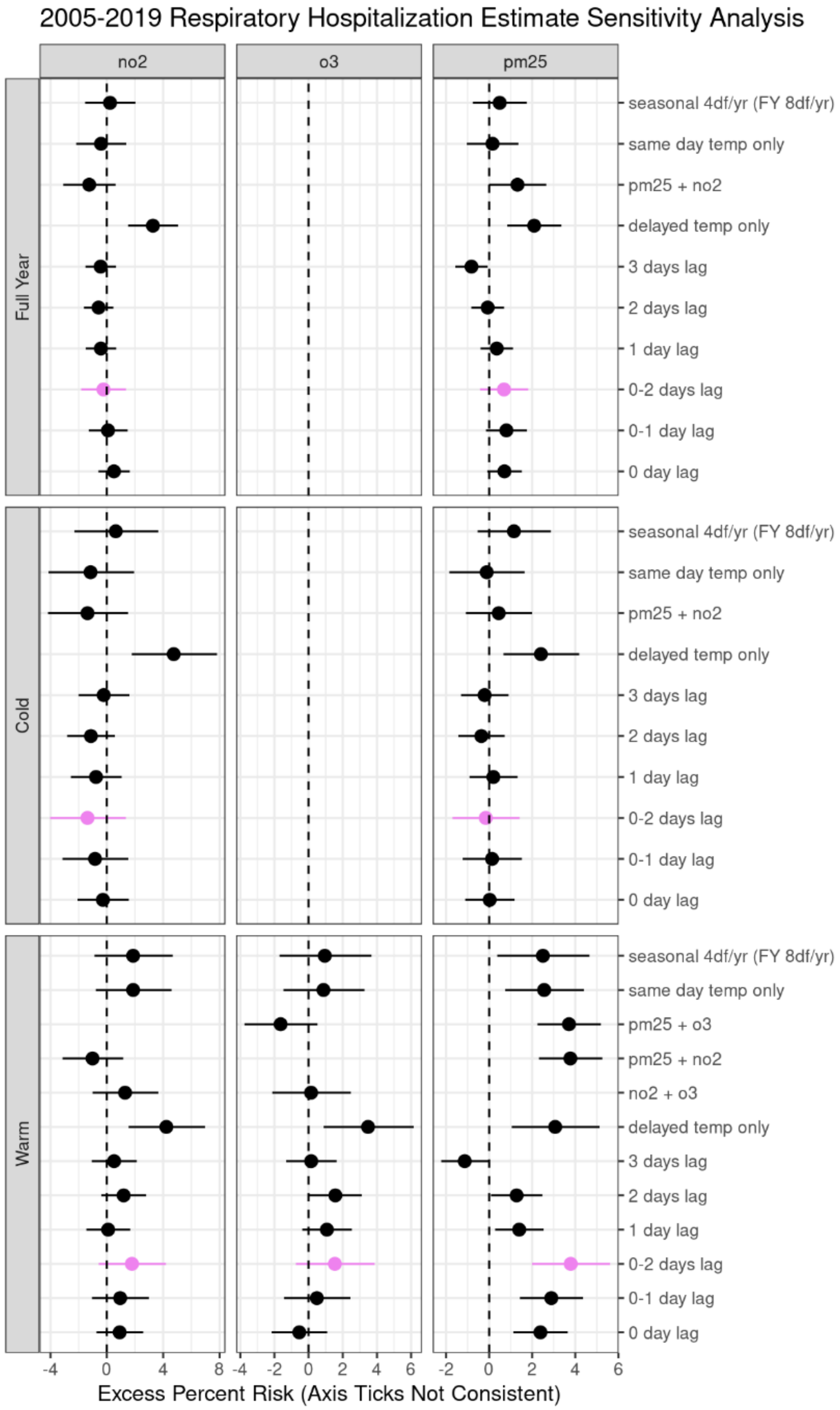

Supplemental Information for Changes in respiratory and cardiovascular morbidity air pollution risk and attributable burden in New York City Ariel Spira-Cohen, Rebecca Goldberg, Sarah Johnson, Masha Pitiranggon, Kazuhiko Ito

Asthma warm season model sensitivity analysis

Due to the spring pollen peak, midsummer trough, and school opening peak for asthma morbidity, asthma models were evaluated with different time periods for the warm season. These time periods include: 1) May – Sept (main model), 2) June – Sept, 3) May – Aug, 4) June – Aug. Note that June-Aug models excluded high asthma ED visit count days by design.

Analyzing for different time periods showed that including May increased the risk in the NO_2 and Ozone models, and decreased the risk in the $PM_{2.5}$ models (most visible in the hospitalization models). Including or excluding September did not have a consistent effect on the risk estimates. Risk estimates for the June-Aug months generally had wider confidence intervals due to the lower counts during the peak of summer and widened the already wide confidence intervals for the hospitalization outcomes. In all models, the risk estimates were comparable or larger than the corresponding cold season estimates and confidence intervals overlapped with full year risk estimates for $PM_{2.5}$ and NO_2. Our sensitivity analysis showed a greater impact of the month of May than September on warm season NO_2 and Ozone effects on asthma morbidity, which is supported by research showing some evidence of an interaction effect on asthma of these pollutants with outdoor aeroallergens both mechanistically[4] and in epidemiological models.[5]

Figure S-12A & Figure S-12B:

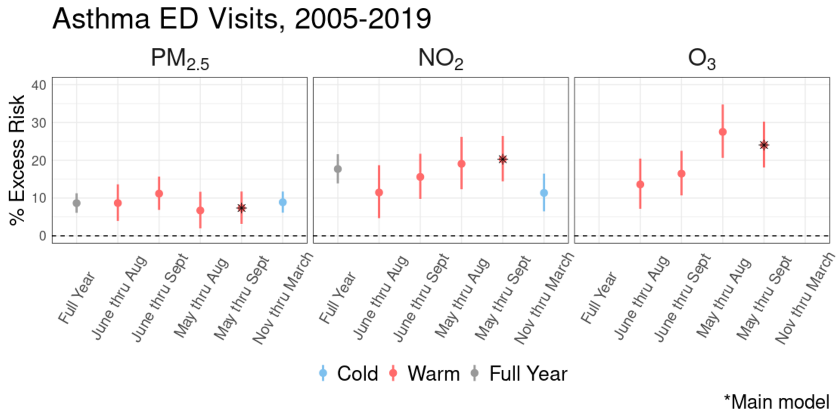

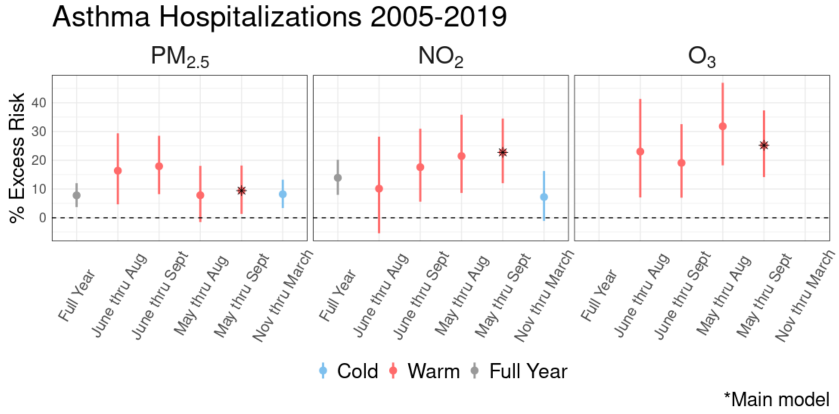

Supplemental Information for Changes in respiratory and cardiovascular morbidity air pollution risk and attributable burden in New York City Ariel Spira-Cohen, Rebecca Goldberg, Sarah Johnson, Masha Pitiranggon, Kazuhiko Ito

Analysis of NO_2 and O_3 with CVD hospitalizations in the warm months

The warm season percent excess risks (PER) for NO_2 and CVD hospitalizations (Figure S-2) and ozone and CVD hospitalizations (Figure 3) in the earlier years were consecutively negative (i.e., apparent "protective" effect) and then increased to positive in the later years. The negative

PER's and their increasing trends to positive associations were counterintuitive, so we investigated potential cause(s). First, we examined the correlation of regression coefficients between these pollutants and covariates and found that the same-day temperature term was moderately negatively correlated with regression coefficients for NO_2 (i.e., -0.62 in the earliest 5-yr window and weakened to -0.45 in the most recent period, when temperature was fitted as a linear term) and O_3 (i.e., -0.67 in the earliest 5-yr window and generally unchanged over time, with -0.65 in the most recent period). The negative correlations of regression coefficients between these pollutants and same-day temperature indicate that their associations with CVD hospitalizations are "shared" (i.e., if the estimate for one variable is higher than its true value, the estimate for the other variable would be lower than its true value). The negative correlations of the regression coefficients also indicate instability of these regression coefficients. Therefore, to observe the impact of the same-day temperature adjustment on PER's for NO_2 and ozone, we ran an alternative model without same-day temperature in the rolling 5-year time windows for the warm season, as shown in the supplemental Figure S-9-(A) for NO_2 and (B) for ozone, and compared the results with those from the main model with same-day temperature. The PER's in models without adjustment for same-day temperature are either null or weakly positive in the earlier years when the PER's were negative in the model with adjustment for the same-day. In the later years, the PER's from the model without same-day temperature also became positive but generally were larger than those from the main model for both pollutants.

Because a negative correlation of regression coefficients for two predictors tends to reflect their positive correlation, we also examined pairwise correlations of all the pollutants and temperature, both the same-day and the average of lag 1 through 3 days, in the same 5-year rolling time windows, as shown in the supplemental Figures S-9A & S-9B. The most notable changes in correlation during the 20-year period were between NO_2 and both the same-day and the average of lag 1-3 days temperature variables, both of which declined starting around 2006-2010. The correlations between NO_2 and the same-day temperature declined from about 0.3 in the earlier years to less than 0.1 in the later years. NO_2's correlations with the average of lag 1-3 days temperature also declined from around 0 to less than -0.2. These patterns are consistent with the declining contribution from vehicle-related NO_2 and resulting larger relative contributions from building burning oil, shifting the seasonality of NO_2 from summer-peaking to winter-peaking over the 20-year period. The weakening of correlation between NO_2 and the same-day temperature is consistent with the weakening of the negative correlation of their regression coefficients and corresponding changes in PER's from negative to positive in the full model. However, the correlation between ozone and the same-day temperature (positive, ~0.6) and the correlation of their regression coefficients (negative, ~-0.65) remained the same during the study period and thus cannot explain the change in PER's for ozone and CVD hospitalizations.

In summary, the negative warm season PER's observed for the associations between NO_2 and CVD hospitalizations and ozone and CVD hospitalizations in the earlier years were

induced by the adjustment for same-day temperature due to its positive correlation with these pollutants. Without the adjustment for the same-day temperature, the PER's for NO_2 and ozone for CVD hospitalizations in the earlier years are null or slightly positive. While the collinearity of these pollutants with the same-day temperature makes it challenging to interpret these PER's in a few cases such as these, we used the main model with adjustment for same-day temperature to conservatively estimate the pollution effects.

Figure S-13A and Figure S-13B. Percent excess risks in rolling 5-year time windows

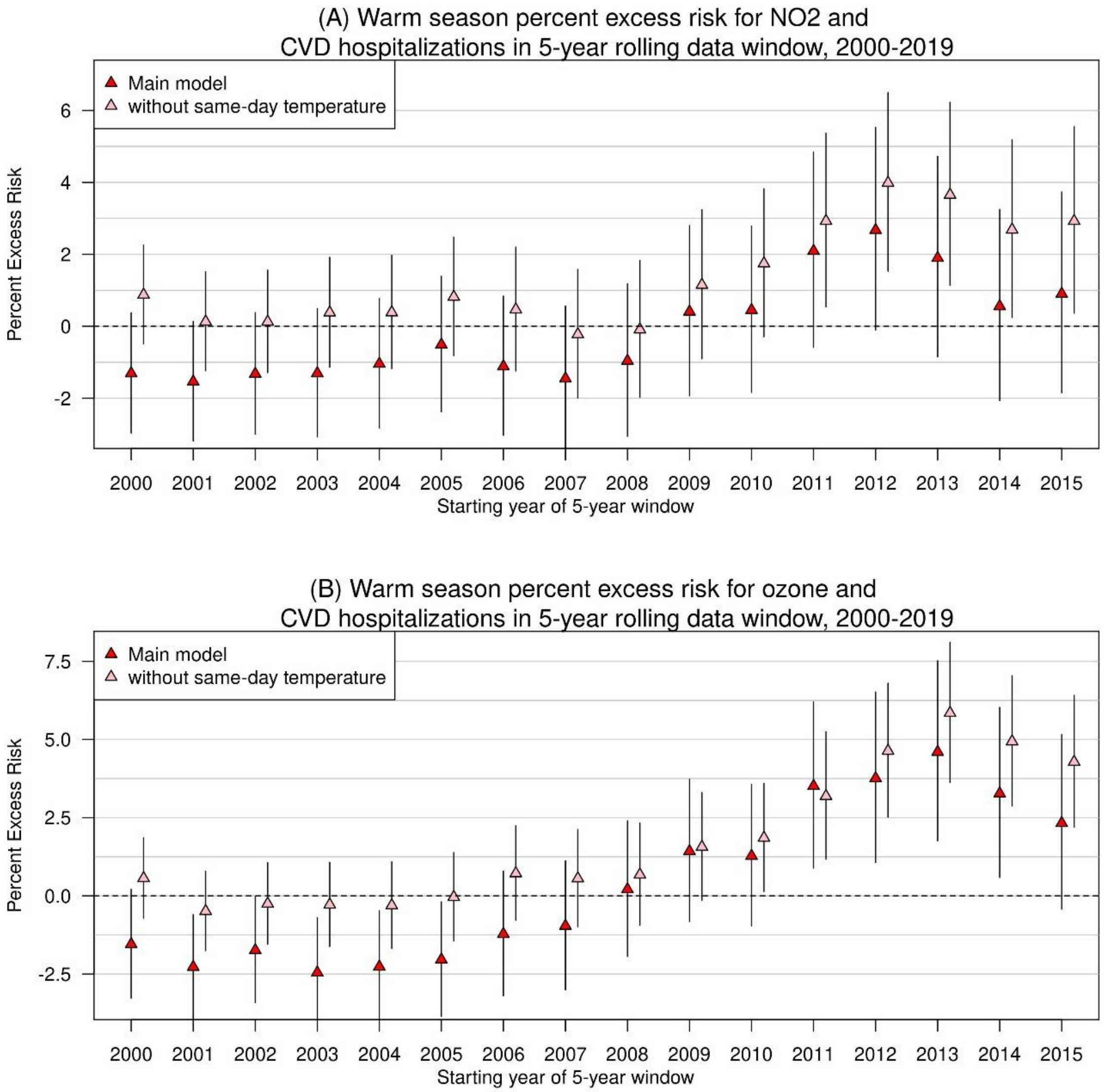

Supplemental Information for Changes in respiratory and cardiovascular morbidity air pollution risk and attributable burden in New York City Ariel Spira-Cohen, Rebecca Goldberg, Sarah Johnson, Masha Pitiranggon, Kazuhiko Ito

Figure S-14. Trend in pairwise correlation among air pollutants and temperature variables in the 5-year moving time windows. “Temp0”is the same-day temperature and “Temp13” is the average of lag 1 through 3 days temperature.

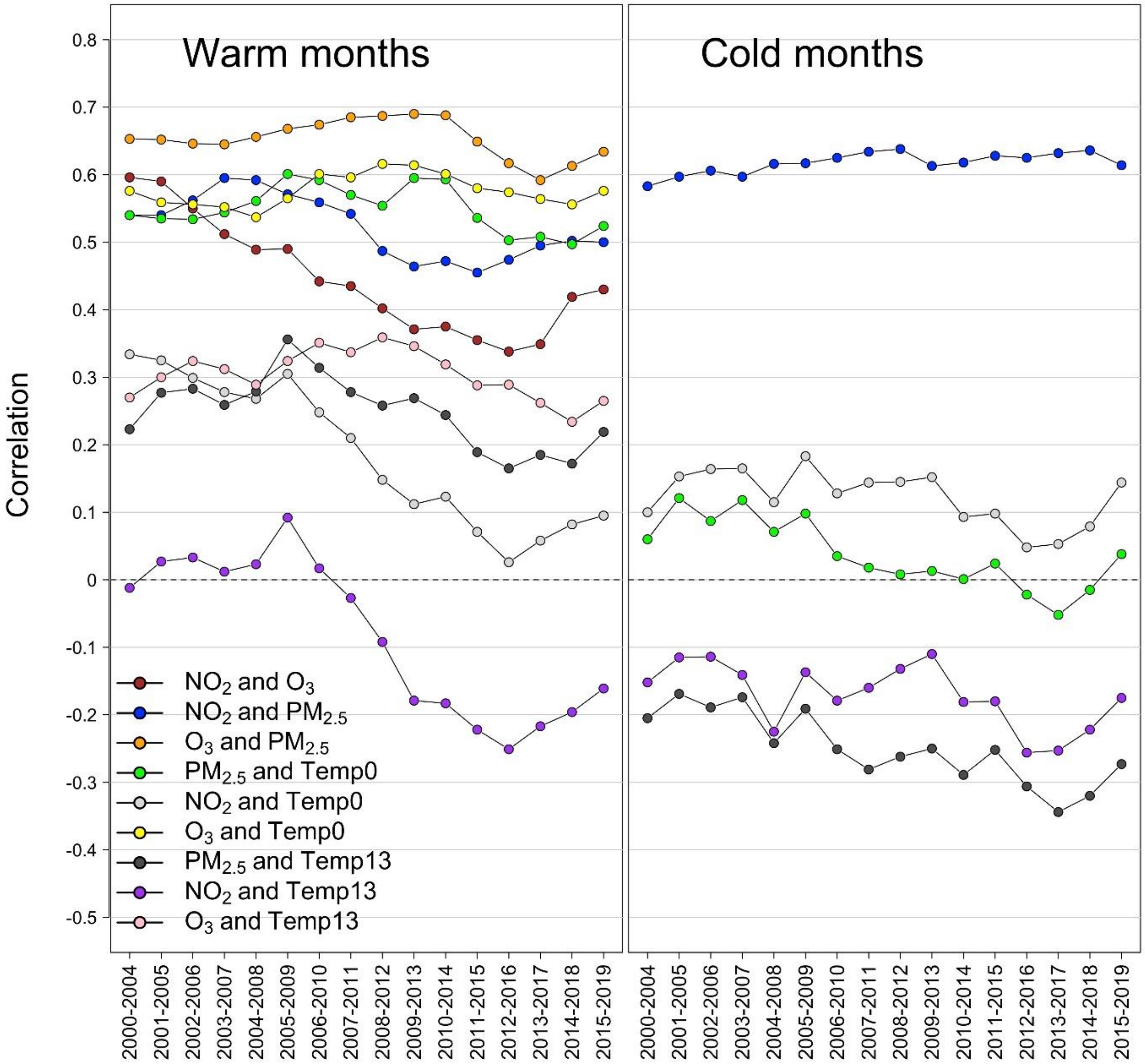

Supplemental Information for Changes in respiratory and cardiovascular morbidity air pollution risk and attributable burden in New York City Ariel Spira-Cohen, Rebecca Goldberg, Sarah Johnson, Masha Pitiranggon, Kazuhiko Ito

BENMAP versus attrdl comparison

We estimated the burden of exposures to $PM_{2.5}$, nitrogen dioxide, and ozone based on data from EPA's Air Quality System (AQS). Pollutant data were limited to those assigned a population exposure monitoring objective. Completeness criteria were applied so that hourly data from a given day were excluded if less than 75% of daily measurements were collected, and any data were excluded if collected from a monitor that did not meet EPA criteria for annual completeness. Data were aggregated to create cold season, warm season, and full-year averages at each EPA monitoring site over the 2005-2009 period. Average concentrations were assigned to each of 216 populated Zip Code Tabulation Areas (ZCTAs) using an averaging approach within BenMAP known as Voronoi Neighbor Averaging (VNA). The VNA algorithm identifies monitors that best surround a point of interest (in this case the centroid of a given ZCTA) then calculates the inverse distance weighted average concentration of the values from these monitors. We ran BenMAP for each season using health incidence data and population estimates for each ZCTA, averaged over the years 2005-2009. Changes in health outcome attributed to air pollution were calculated using health impact functions taking the form:

$$\Delta I = \left(1 - e^{-\beta \Delta X}\right) * P * I_0$$

Where ΔI is the change in the number of health events associated with the air pollutant concentration (ΔX), β is the effect coefficient from an epidemiological study, P is the exposed population, and I_0 is the baseline rate of disease or death. Results from the 216 ZCTAs were summed to produce citywide attributable counts. The effect coefficient used was from the epidemiological models in our study (2005-2019). While the epi model covered the full time period, the 2005-2009 data subset was used to extract the attributable counts to match the time period used in BenMAP. Only pollutant-outcome pairs with overall positive associations were included in this comparison. There were minimal differences in numbers between the two methods.

Table S-3: Comparison of annual average pollution attributable youth asthma ED visits and hospitalizations from single pollutant risk estimates (2005-2019) using BenMAP versus the attrdl function for 2005-2009 period

		BENMAP		ATTRDL	
SEASON	**Pollutant**	**Average AC**	**95% CI**	**Average AC**	**95% CI**
**ED VISITS**					
**WARM**					
	O_3	**2336**	**(1852, 2773)**	**2187**	(1734, 2637)
	NO_2	**2130**	**(1595, 2609)**	**2008**	(1507, 2503)
	$PM_{2.5}$	**817**	**(357, 1238)**	**694**	(307, 1084)
**COLD**					
	NO_2	**1714**	**(1017, 2344)**	**1663**	(987, 2327)

	$PM_{2.5}$	**1272**	**(895, 1622)**	1238	(877, 1607)
**FULL YEAR**					
	NO_2	**5427**	**(4406, 6365)**	**5193**	(4225, 6175)
	$PM_{2.5}$	**2580**	**(1851, 3261)**	**2383**	(1721, 3069)
**HOSPITALIZATIONS**					
**WARM**					
	O_3	**391**	**(241, 519)**	**351**	**(211, 482)**
	NO_2	**375**	**(217, 508)**	**346**	**(198, 483)**
	$PM_{2.5}$	**164**	**(22, 288)**	**135**	**(16, 248)**
**COLD**					
	NO_2	**178**	**(-36, 361)**	**173**	(-35, 366)
	$PM_{2.5}$	**183**	**(77, 279)**	**180**	(77, 283)
**FULL YEAR**					
	NO_2	**708**	**(426, 960)**	**668**	(401, 932)
	$PM_{2.5}$	**371**	**(180, 547)**	**340**	(165, 518)

Table S-4: Comparison of annual average pollution attributable adult cardiovascular ED visits and hospitalizations from single pollutant risk estimates (2005-2019) using BenMAP versus the attrdl function for 2005-2009 period

		BENMAP		ATTRDL	
SEASON	**Pollutant**	**Average AC**	**95% CI**	**Average AC**	**95% CI**
**ED VISITS**					
**COLD**					
	NO_2	**447**	**(37, 834)**	**434**	(52, 828)
	$PM_{2.5}$	**369**	**(144, 582)**	**373**	(155, 597)
**FULL YEAR**					
	NO_2	**656**	**(72, 1211)**	**633**	(93, 1194)
	$PM_{2.5}$	**443**	**(86, 786)**	**447**	(101, 807)
**HOSPITALIZATIONS**					
**COLD**					
	NO_2	**802**	**(284, 1289)**	**781**	(298, 1278)
	$PM_{2.5}$	**411**	**(129, 1072)**	**417**	(143, 701)
**FULL YEAR**					

	NO_2	**1539**	**(818, 2224)**	**1493**	(823, 2189)
	$PM_{2.5}$	**651**	**(210, 1072)**	**660**	(232, 1105)

Table S-5: Comparison of annual average pollution attributable older adult respiratory ED visits and hospitalizations from single pollutant risk estimates (2005-2019) using BenMAP versus the attrdl function for 2005-2009 period

		BENMAP		ATTRDL	
SEASON	**Pollutant**	**Average AC**	**95% CI**	**Average AC**	**95% CI**
**ED VISITS**					
**WARM**					
	O_3	**307**	**(-126, 712)**	**306**	(-121, 717)
	NO_2	**289**	**(-181, 728)**	**273**	(-173, 700)
	$PM_{2.5}$	**376**	**(53, 681)**	**366**	(47, 668)
**HOSPITALIZATIONS**					
**WARM**					
	O_3	**224**	**(-171, 592)**	**224**	**(-167, 597)**
	NO_2	**272**	**(-155, 670)**	**258**	**(-149, 645)**
	$PM_{2.5}$	**578**	**(297, 841)**	**568**	**(289, 829)**

Trends in pollution attributable burden using 5-year moving window models

The five-year moving time window models were used to estimate the attributable burden during each of these five-year periods. Due to the windows having overlapping years, to analyze the trend the annual average was calculated from each five-year model. This method differs from the main result because calculating the attributable burden using a subset of the data by year would have produced multiple yearly estimates from each five-year model. For the most recent time-period, for example, the number of pollution-attributable ED visits and hospitalizations reflects the annual average across the 2015-2019 period using the PER for that same period. This method produced more variation in burden over time than the results of the main analysis, but many outcomes still showed declines.

Figure S-15: Trends in pollution-attributable ED visits and hospitalizations for A) NO_2, B) $PM_{2.5}$ and C) O_3

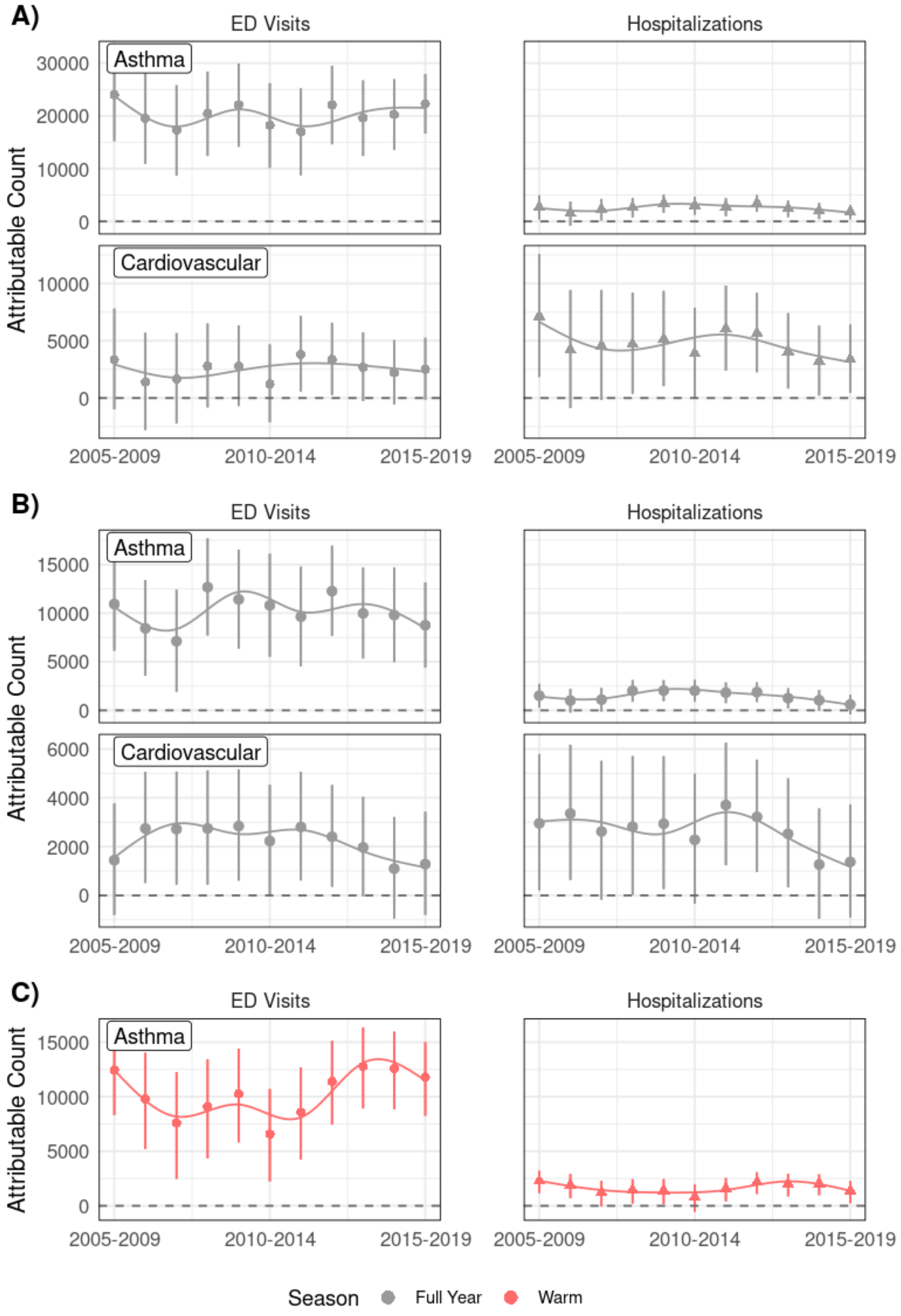

Citations for Supplemental Material:

Supplemental Information for Changes in respiratory and cardiovascular morbidity air pollution risk and attributable burden in New York City Ariel Spira-Cohen, Rebecca Goldberg, Sarah Johnson, Masha Pitiranggon, Kazuhiko Ito

1. (USEPA) USEPA. Air Quality System (AQS). United States: United States Environmental Protection Agency.
2. Ito K, Thurston GD, Silverman RA. Characterization of PM2.5, gaseous pollutants, and meteorological interactions in the context of time-series health effects models. *J Expo Sci Environ Epidemiol* 2007;**17 Suppl 2**:S45-60.
3. Schwartz J. The Distributed Lag between Air Pollution and Daily Deaths. *Epidemiology* 2000;**11**(3).
4. Sedghy F, Varasteh AR, Sankian M, Moghadam M. Interaction Between Air Pollutants and Pollen Grains: The Role on the Rising Trend in Allergy. *Reports of Biochemistry & Molecular Biology* 2018;**6**.
5. Lam HCY, Jarvis D, Fuertes E. Interactive effects of allergens and air pollution on respiratory health: A systematic review. *Sci Total Environ* 2021;**757**:143924.
